# Quantitative Variables Derived from the Electroencephalographic Signal to Assess Depth of Anaesthesia in Animals: A Narrative Review

**DOI:** 10.3390/ani15152285

**Published:** 2025-08-05

**Authors:** Susanne Figueroa, Olivier L. Levionnois, Alessandro Mirra

**Affiliations:** 1 Division of Anaesthesiology and Pain Therapy, Vetsuisse Faculty, University of Bern, 3012 Bern, Switzerland; susanne.koroschetz@gmx.at (S.F.); alessandro.mirra@umontreal.ca (A.M.); 2 Tierarztpraxis mag.med.vet. Daniel Rieder, 7121 Weiden am See, Austria; 3 Department of Clinical Sciences, Faculty of Veterinary Medicine, University of Montreal, Saint-Hyacinthe, QC J2S 2M2, Canada

**Keywords:** animal anaesthesia, depth of anaesthesia, electroencephalography, nociception, quantitative EEG variables, veterinary monitoring

## Abstract

Accurately assessing the depth of anaesthesia in animals remains a challenge, as traditional monitoring methods fail to capture subtle changes in brain activity. This review aimed to systematically map and critically evaluate the range of quantitative variables derived from electroencephalography (EEG) used to monitor sedation or anaesthesia in live animals, excluding laboratory rodents, over the past 35 years. Studies were identified through comprehensive searches in major biomedical databases (PubMed, Embase, CAB Abstract). To be included, studies had to report EEG use in relation to anaesthesia or sedation in living animals. A total of 169 studies were selected after screening and data extraction. Information was charted by animal species and reported EEG-derived variables. The most frequently reported variables were spectral edge frequencies, spectral power metrics, suppression ratio, and proprietary indices, such as the Bispectral Index. Methodological variability was high, and no consensus emerged on optimal EEG measures across species. While EEG-derived quantitative variables provide valuable insights, their interpretation remains highly context-dependent. Further research is necessary to refine these methods, explore variable combinations, and improve their clinical relevance in veterinary medicine.

## Simple Summary

Assessing anaesthesia depth in animals during medical procedures is important for their safety and welfare. However, current monitoring methods fail to detect subtle changes in brain activity. This review examined published research from the past 35 years to identify and summarise how electrical brain signals, recorded using electroencephalography (EEG), have been used to assess anaesthesia effects in various animal species, excluding laboratory rodents. The review found that most studies focused on measuring specific EEG features, such as the amount of electrical activity at different frequency bands, the proportion of time the brain is very quiet, or combined indices generated by commercial monitors. However, the selection and interpretation of these measurements varied widely across studies and animal species. As no standardised approach currently exists, further research is needed to refine these methods. Combining different EEG measurements may improve accuracy in monitoring anaesthetic depth in animals. Improved EEG-based assessment could support veterinarians in delivering safer and more effective care during surgical and diagnostic procedures.

## 1. Introduction

Despite significant advances in understanding the neurophysiological mechanisms of anaesthesia, accurately assessing depth of anaesthesia (DoA) remains difficult [1]. Conventional tools—such as clinical scoring systems and autonomic reflex monitoring—typically lack sensitivity to subtle transitions between anaesthetic states [2,3]. DoA is inherently multifaceted. It involves components such as consciousness suppression (hypnosis), muscle relaxation, immobility, suppression of nociceptive responses, and disruption of physiological stability. Among these, evaluating the extent of hypnosis or loss of consciousness is especially relevant when titrating an anaesthetic dose.

Electroencephalography (EEG), which records cortical electrical activity, emerged nearly 90 years ago as a promising method for quantifying DoA in humans [4,5]. However, its application in animal studies remains inconsistent. Differences in methodologies and species-specific features limit its reliability, with no consensus regarding the most reliable metrics for correlating with anaesthetic depth.

In human anaesthesia, integrative EEG indices like the Bispectral Index (BIS) and entropy-based measures have improved real-time DoA monitoring [6]. Recent developments, including spectrogram visualisation [4] and machine learning algorithms [7], offer new opportunities for real-time detection of subtle EEG changes over time (power distribution in the frequency domain). Yet, their adoption in animal research is still emerging.

Given these challenges, a comprehensive overview of the EEG-derived variables investigated in anaesthetised animals is warranted. This review is intended to systematically map the landscape of quantitative EEG variables employed in animals under anaesthesia, highlighting trends in their use and evaluating the strength of evidence supporting their association with DoA. For this purpose, the process of a scoping review was targeted, and the following research question was formulated: Which EEG-derived parameters have been reported in the literature in relation to the evaluation of the depth of anaesthesia in animals? By synthesising data across species and experimental paradigms, we aim to identify the most widely reported metrics and potential methodological inconsistencies. The findings may help researchers select suitable EEG biomarkers and improve anaesthesia monitoring in veterinary contexts.

## 2. Materials and Methods

This review was conducted in accordance with the PRISMA-ScR (Preferred Reporting Items for Systematic Reviews and Meta-Analyses extension for Scoping Reviews) checklist. The checklist was used to ensure a structured, transparent approach and to systematically evaluate the topic.

### 2.1. Search Strategy (Stage 1)

A systematic literature search was conducted in April 2023, beginning with pilot searches to refine the keywords. The final search was performed on 12 April using the Ovid search engine to access three databases: PubMed, including MEDLINE records for biomedical literature; Embase (Excerpta Medica Database), which covers biomedical and pharmacological research; and CAB Abstracts, which focusses on animal health and veterinary sciences. The aim was to identify studies investigating the use of EEG as a monitoring tool to assess depth of anaesthesia in animals. To exclude studies using outdated signal acquisition methods, the search was limited to articles published from 1 January 1990, onward. No language restrictions were applied at this stage.

The search terms combined keywords related to EEG, anaesthesia and sedation, and animals. Full search strings are reported in the Supplementary Material (Tables S1–S3). Search results were imported into Mendeley Reference Manager for organisation and management. Duplicates were systematically removed. All references were then transferred to a Microsoft Excel spreadsheet and sorted alphabetically by the first author’s surname. Each reference was identified by the first author’s name, year of publication, ISSN number, and article title to facilitate further screening.

### 2.2. Screening and Eligibility (Stages 2 and 3)

A data-charting form was jointly developed by the three primary investigators using Microsoft Excel. An initial test phase was carried out to ensure the form was adequate. References obtained from stage 1 were screened for relevance in two subsequent stages. Stage 2 involved screening titles and abstracts, and stage 3 consisted of a full-text review. Articles were assessed using predefined inclusion and exclusion criteria. Each criterion was assigned a status of “yes”, “no”, or “unclear”. If the status was unclear, the article was retained for further review in the next stage. 

During stage 2, the following screening criteria were applied:

- Original Research: Only articles identified as an original research type were included. Articles such as opinion papers and reviews were excluded.
- Animal: Only articles investigating animals were included. Studies involving human participants or electronic models were excluded.
- In vivo: Only studies on live animals were included.
- EEG: Only studies reporting the use of electroencephalography as one of the main outcomes were included.
- Anaesthetic/sedative drug administered: Only studies reporting the use of an anaesthetic and/or a sedative agent were included.

During stage 3 (full-text screening), the following criteria were added:

- Publication language: Only main full text available in English were kept included. Others were excluded.
- Relation between EEG and anaesthesia/nociception: Only studies where the EEG signals (or EEG-derived variables) were collected in relation to anaesthesia, sedation, or (anti-)nociception were kept. Others were excluded.

To ensure consistency during screening, a calibration phase was conducted at the beginning of stage 2. All three investigators independently reviewed the same 100 articles, which allowed refinement of the screening strategy. The remaining screening was primarily performed by one investigator (S.F.). Any uncertain cases were reviewed independently by the other two investigators (O.L.L., A.M.), and final decisions were made through consensus.

### 2.3. Data Extraction

For each eligible study, all reported EEG-derived quantitative variables were recorded. Additional information was extracted on the research focus (anaesthesia, sedation, or antinociception) and the animal species studied. The EEG variables were then grouped into subcategories to facilitate presentation and discussion.

### 2.4. Critical Appraisal

Following study selection and data extraction, all investigators conducted a subjective critical appraisal of the findings. Each paper was assigned a level of evidence based on the study design, following the hierarchy of the evidence pyramid [8] (from 1 to 6—1: Case series/Case report, 2: Observational study, 3: Case control study, 4: Cohort study, 5: Randomised controlled trial, 6: Systematic review/Meta-analysis). This evaluation focused on how EEG-derived variables were reported in relation to monitoring DoA or nociception, even if this was not the primary outcome of the study.

In addition, a synthesis of the main findings from the literature is presented in the discussion. The aim of this narrative synthesis was to identify trends in the use of EEG-derived variables across species and experimental contexts. Methodological inconsistencies were also highlighted.

## 3. Results

**Figure 1:**
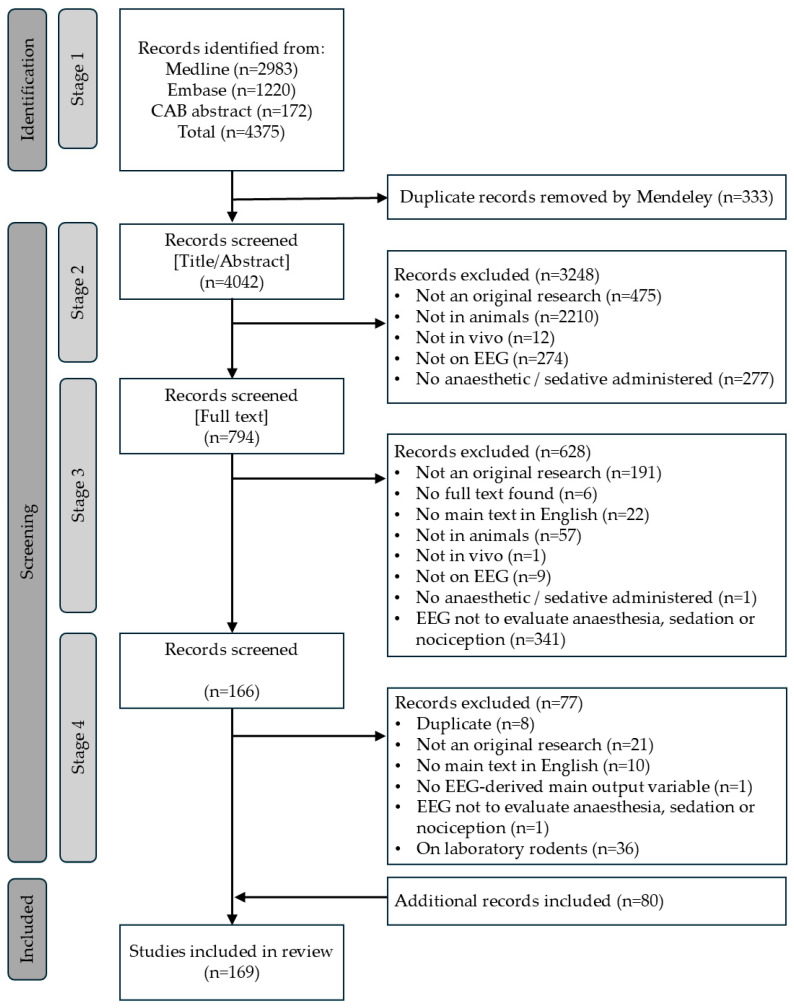
Flow diagram of the systematic selection process for references reporting the use of EEG-derived quantitative variables to estimate depth of anaesthesia in animals.

The flow diagram of the selection process is presented in Figure 1.

During stage 1, an initial pool of 4375 references were retrieved through the search strategy. Following duplicates removal, 4042 references remained. At stage 2 (abstract screening), a total of 794 references were retained. Following full-text screening in stage 3, a total of 166 articles met the inclusion criteria and were finally kept.

Subsequent review by all authors revealed inconsistencies, including remaining duplicates and erroneously included references such as conference abstracts or articles in languages other than English. As a result, a fourth screening stage (stage 4) was added. In this stage, 77 references were removed, leaving a final selection of 89 articles. At the same time, additional relevant studies were identified by the authors through domain knowledge, incidental findings, or backward citation searches. These had likely been missed during stage 1 or were wrongly excluded. Repeating the full screening process was deemed too time-consuming. Therefore, the decision was made to include these additional references to the best of our ability. However, this adjustment meant that the systematic nature of the review could no longer be fully preserved, and the study shifted toward a narrative review. Many of the newly identified references involved laboratory rodents (e.g., rats, mice, gerbil). To maintain focus and consistency, all studies involving laboratory rodents were excluded from the final review. Detailed figures are presented in Figure 1 and Table S4.

The final selection comprises 169 references [3,6,9,10,11,12,13,14,15,16,17,18,19,20,21,22,23,24,25,26,27,28,29,30,31,32,33,34,35,36,37,38,39,40,41,42,43,44,45,46,47,48,49,50,51,52,53,54,55,56,57,58,59,60,61,62,63,64,65,66,67,68,69,70,71,72,73,74,75,76,77,78,79,80,81,82,83,84,85,86,87,88,89,90,91,92,93,94,95,96,97,98,99,100,101,102,103,104,105,106,107,108,109,110,111,112,113,114,115,116,117,118,119,120,121,122,123,124,125,126,127,128,129,130,131,132,133,134,135,136,137,138,139,140,141,142,143,144,145,146,147,148,149,150,151,152,153,154,155,156,157,158,159,160,161,162,163,164,165,166,167,168,169,170,171,172,173,174,175], as shown in Table S4. Subdivisions according to research topic, animal species, and material reported within the final selection are provided in Table 1.

**Figure 2:**
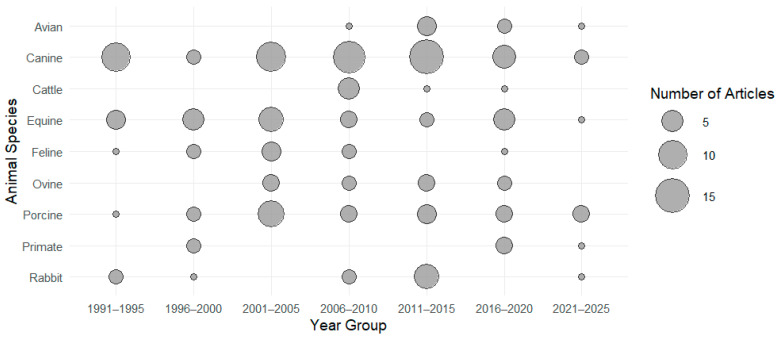
Distribution per animal species of research articles investigating the use of EEG-derived quantitative variables to evaluate depth of anaesthesia over the years (publication date).

**Figure 3:**
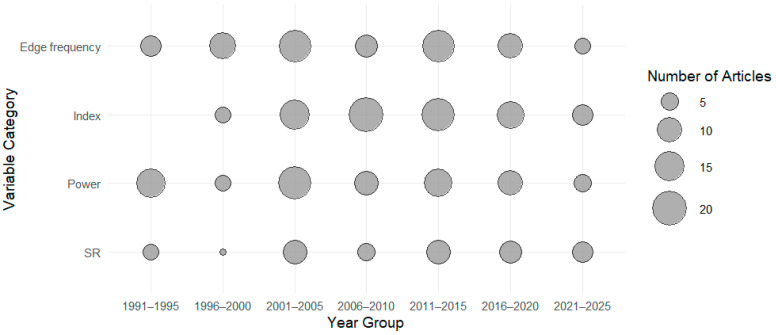
Distribution per categories of research articles investigating the use of EEG-derived quantitative variables to evaluate depth of anaesthesia over the years (publication date). Edge frequency: Articles reporting on the use of the dominant EEG frequency to evaluate DoA; Index: Articles reporting on the use of an EEG Index of DoA; Power: Articles reporting on the use of the EEG power to evaluate DoA; SR: Articles reporting on the use of the EEG suppression ratio to evaluate DoA.

The distribution of animal species investigated over time is shown in Figure 2. The distribution of research articles according to categories of EEG-derived variables (e.g., suppression ratio, power-based variables, frequency-based variables, index, etc.) is presented in Figure 3. (*) One paper reported the use of both porcines and caprines.

The level of evidence for each article is presented in Table S4. A small number of studies reported EEG-derived variables as a secondary aim (*n* = 16, corresponding to 9%). Most of these were considered observational studies (*n* = 15), and one was considered a randomised controlled trial. The remaining studies reported EEG-derived variables as the primary aim (*n* = 153, corresponding to 91%). Among these, the majority were judged observational studies (*n* = 86, corresponding to 51%), followed by case control studies (*n* = 58, corresponding to 34%), and a smaller number of randomised controlled trials (*n* = 9, corresponding to 5%).

The list of EEG-derived variables investigated included in the review is presented in Table 2.

## 4. Critical Appraisal and Discussion

Over the last 35 years (1990–2023), at least 169 publications (mean 5 ± 3 per year) were identified that investigated EEG-derived quantitative variables in the evaluation of DoA in animals, excluding laboratory rodents. The most commonly reported EEG-derived variables were spectral edge frequencies (44% of studies), spectral power and related ratios (43%), suppression ratio (26%), Bispectral Index (BIS; 34%), entropy (5%), and Patient State Index (PSI; 4%). The publications were relatively evenly distributed over the years, with no clear trend toward increased research activity during this period.

### 4.1. Limitations

This review has several limitations. The initial goal was to conduct a systematic scoping review, but this was not possible due to methodological errors encountered during the process. Although it is unlikely that many relevant studies were missing, the exact number of publications to be included cannot be guaranteed. All screened publications meeting the selection criteria were included; no paper was excluded arbitrarily or due to personal preferences.

During the screening process, research articles on EEG in laboratory rodents were excluded. All animal species were considered at first, but studies involving rats and mice accounted for most of the irrelevant results. This was likely due in part to difficulties distinguishing, based solely on abstracts (stage 2), between studies focused on neurological diseases (e.g., epilepsy) and those evaluating depth of anaesthesia. After abstract review (stage 2), the number of publications on laboratory rodents was approximately twice that of studies involving dogs. Including these rodent studies would likely have added more EEG-derived variables, but most of these may have been less relevant to clinical practice.

Another limitation is that only studies published after 1990 were included. This choice was intentional, as studies before this date likely used outdated EEG technology, including older electrodes, amplifiers, data storage methods, and computational techniques.

Critical appraisal of systematic reviews usually involves assessing the certainty of evidence and the level of bias. This helps determine how much confidence can be placed in the results reported by the selected literature [176]. However, the GRADE system was not applicable to the present review because the main goal was not to produce a single quantitative outcome. Quality grading of each article using established tools [177,178], such as the Critical Appraisal Skills Programme (CASP) checklist or the Joanna Briggs Institute (JBI) tool, proved difficult and unreliable. Most of the included papers did not directly test whether a specific EEG-derived variable could distinguish between different DoA or nociceptive states. Instead, they reported a range of variables applied to a variety of conditions. For these reasons, the review took a broad approach, aiming mainly to collect and summarise the EEG-derived variables reported, their methodology, and the contexts of their use. Only cautious conclusions can be drawn. Most studies were observational in design, which indicates a low overall quality of evidence. As a result, the conclusions presented in the following discussion should be interpreted with caution.

### 4.2. Animal Species

Dogs, horses, and pigs were the most frequently studied animal species. In contrast, studies involving cattle more often focused on the depth of anaesthesia in the context of slaughter procedures. These studies aimed to assess the timing and quality of loss of consciousness when comparing methods of killing.

### 4.3. Suppression Ratio

The suppression ratio was reported in 26% of the studies, and 75% of these (*n* = 32) used commercial DoA devices, primarily the BIS monitor. The BIS device calculates the SR as the percentage of the preceding 63 s period during which EEG activity is suppressed, typically defined as an amplitude within ± 5 microvolts for at least 0.5 s [179,180]. The SR is usually updated every 1–2 s. While other commercial monitors likely use similar methods, the precise algorithms remain proprietary and are not publicly disclosed. Several studies used different definitions for suppression. For example, Martoft et al. applied the same threshold as the BIS [51], Rampil et al. used a stricter definition (<5 microvolts peak-to-peak) [12], and McIlhone et al. defined suppression as an EEG amplitude less than 12.5% of baseline [134,156]. Species-specific adjustments have also been proposed. For instance, Koyama et al. found that a threshold of <±2.25 microvolts for at least 0.35 s might be more appropriate in sevoflurane-anaesthetised dogs [158].

Six studies assessed SR qualitatively, using a simple binary classification (active/suppressed) based on direct visual observation of the EEG [66]. This approach tends to yield higher SR values [180] and allows earlier detection of suppression compared to automated algorithms [181]. Although the American Clinical Neurophysiology Society guidelines are recommended for visual analysis [182], quantitative SR assessment by visual inspection has not been reported in anaesthetised animals.

SR is mainly associated with deep levels of anaesthesia, but its numerical value is rarely analysed in detail. Some authors have compared SR values across different anaesthetic planes [34,105,116,173]. Haga et al. investigated the concentration of inhalant anaesthetic required to increase SR above 20%, in relation to the minimum alveolar concentration in pigs and goats [112]. In many studies, SR increased rapidly and discriminated poorly between varying DoA states. A pharmacokinetic model in pigs showed a very steep, sigmoidal relationship between SR and the propofol infusion rate [3]. However, in dogs, SR correlated better with anaesthetic planes than the Cerebral State Index (CSI), although it increased only at deeper levels [126]. Additionally, SR values differed between clinically assessed stages of DoA in horses [171] and chickens [91].

The suppression ratio is often referred to as the burst suppression ratio (BSR). This term likely comes from early descriptions of EEG patterns during deep anaesthesia, where high-voltage spikes alternate with flat, suppressed periods (as seen with agents such as avertin, ether, or barbiturates) [183,184]. Recent studies have focused mainly on measuring the amount of suppressed EEG. In contrast, characteristics of the active EEG segments (bursts), especially how they vary with different species or drugs, are still poorly understood. For example, studies in rabbits have shown that the amplitude and duration of bursts can differ between propofol and isoflurane anaesthesia [25]. The suppression ratio is the most reported time domain EEG variable. It is easy to calculate, and its use has been established for many decades. However, SR remains under-investigated and may not have revealed its full potential for assisting in the evaluation of DoA. In humans, higher SR values have been linked to poor quality of anaesthetic recovery, as well as post-operative delirium [185]. So far, no similar investigations have been conducted in this domain in veterinary species.

In summary, the standard definition of the suppression ratio is a practical tool for detecting deep levels of anaesthesia. However, more quantitative approaches with species-specific adjustments and defined cut-off values are still needed.

### 4.4. Total Power

The EEG signal recorded in the time domain is highly fluctuating and stochastic [179]. However, it exhibits recognisable patterns in amplitude and frequency across different depths of anaesthesia [4]. To quantify EEG modulation in the frequency domain, a discrete Fast Fourier Transform (FFT) is applied. This method produces a spectrum that represents power (in μV^2^/Hz or dB) as a function of frequency. The EEG spectrum is typically analysed by frequency bands: delta (0.5–4 Hz), theta (4–8 Hz), alpha (8–12 Hz), and beta (12–30 Hz). There are some variations in these divisions, and additional subdivisions have been reported [186]. Frequencies below 0.5 Hz and above 30–35 Hz (gamma) are generally excluded from standard analysis. Other reviews have described how different anaesthetic drugs affect the EEG spectrum [4,186]. In the present review, total power (over 0.5–30 Hz) was reported in 25% of studies. The total power measures the overall EEG energy and is proportional to the squared amplitude. Although non-specific, total power usually increases when the EGG shifts from high-frequency, low-amplitude patterns (seen in the awake state) to low-frequency, high-amplitude activity seen in sedated or anaesthetised states [14,20,28,109,137,144,151,156,187,188]. As anaesthetic depth increases further, both EEG activity and total power decrease, eventually leading to suppression and, at very deep levels, isoelectricity [14,17,20,96,188]. The exact point at which total power changes from increasing to decreasing is inconsistent across studies. It likely depends on the animal species, the anaesthetic agent used, and the experimental conditions.

Some studies suggest that the transition point—when EEG total power shifts from rising to falling with increasing DoA—may occur before the onset of EEG suppression [17,20,188]. A key limitation of total power is that it cannot detect opposing changes in different frequency bands [187]; as a result, it provides little comparative information between bands, which may differ by orders of magnitude [4]. During low-to-moderate DoA, noxious stimulation typically decreases total power, correlating with arousal responses [61,75,86,95,97,106,140,142,154,155,187,189]. However, at deeper levels of anaesthesia, with already reduced total power, the EEG often shows little or no response to noxious stimuli [44,72,189], even if cardiovascular reactions may still occur [44,72]. For example, one study reported a moderate increase in total power during castration in young pigs under halothane anaesthesia [74], suggesting that the effect of noxious stimulation on EEG total power depends on the level of anaesthesia: arousal may cause a shift from high-to-low amplitude when transitioning from moderate-to-light anaesthesia, or from moderate-to-high amplitude when transitioning from deep-to-moderate anaesthesia. Nevertheless, direct evidence for these shifts is still lacking.

In summary, although total power can be useful for comparing EEG responses to specific events such as noxious stimulation or changes in anaesthetic dose, its pharmacodynamic profile is likely to lack both repeatability and specificity. As a result, total power measurements may not provide clinicians with a clear or reliable interpretative tool.

### 4.5. Band Power

Some studies (*n* = 28) reported detailed calculations of absolute power in each frequency band. With increasing DoA, delta and theta power generally rose, while alpha and beta power decreased [14,28,45,53,109]. When the relative power distribution was reported (*n* = 16), expressed as a percentage of total power, the increase in delta and the decrease in beta were often even more pronounced, while the theta and alpha bands tended to remain more stable [51,53,80,88,109,123]. Several studies tracking spatio-temporal EEG changes as anaesthetic depth increased found that the delta band power rose with DoA, then fell again at the deepest levels (similar to total power changes). The theta and alpha bandwidths followed a similar but less pronounced pattern, while beta power steadily declined from high values in the awake state to marked depression at even moderate anaesthetic depths [14,53,123,188]. Because changes in the delta range are more prominent and consistent, some researchers have used it as a primary variable [43].

To better characterise EEG power variations with anaesthetic state, a few studies (*n* = 10) calculated power ratios comparing higher frequencies with delta power. The beta/delta ratio typically showed the greatest decrease as anaesthesia deepened [37,53,123] and tended to rise with noxious stimulation, when changes occurred [18,88,123]. Similarly, some authors combined the alpha and theta power together and compared this sum to delta power to enhance sensitivity to anaesthetic changes [62].

During human propofol anaesthesia, there is a clear shift in EEG alpha activity: frontal alpha power increases, a phenomenon known as anteriorisation [186]. This distinct pattern is well described in humans, where the traditionally posterior alpha rhythm during wakefulness is lost and a new, coherent frontal alpha emerges during anaesthesia. Increased frontal alpha power is typically seen at moderate anaesthetic depth, and lower alpha power has been associated with a poorer recovery quality [190]. To date, these specific alpha dynamics have not been reported in animals [188].

One study highlighted that distinct frequency components within standard EEG bands may behave differently [109]: for example, during an alfaxalone bolus in dogs, theta band power (4–7 Hz) overall remained stable, but power actually decreased at 4–5 Hz and increased at 6–7 Hz [109]. This finding suggests that more refined analysis—beyond standard EEG bandwidth divisions—may be warranted.

Additionally, a study introduced a Relative Power Index, calculated as a weighted sum of the relative powers across all five dominant bands, which demonstrated strong predictive performance for anaesthetic depth compared to other variables [132].

Collectively, these findings indicate that, despite decades of investigation, band-specific power dynamics contain further untapped information [191]. More research is needed, as these variables show high potential for quickly and reliably distinguishing among anaesthetic states and could assist clinicians in practice.

### 4.6. Spectral Edge Frequency

Spectral edge frequency (95%, SEF95) and median frequency (50%, SEF50 or MEF) were the most frequently reported quantitative EEG variables. Edge frequencies represented the second most common category after dedicated DoA indices. An edge frequency refers to the cut-off value (in Hz) below which a specified percentage of the signal’s total power is contained [192]. Notably, these values are strongly influenced by the settings of band-pass filters.

Edge frequencies serve as surrogates for relative power ratios. For example, a delta/theta-dominant EEG exhibits a lower SEF95 than an alpha/beta-dominant EEG during cortical arousal. However, they are not directly affected by changes in total power. Although a single value cannot fully capture the complex, multimodal structure of the EEG spectrum, edge frequencies can reflect broad changes across different anaesthetic states [22,34,50,123,163,192]. Still, several studies report limited sensitivity of spectral edge frequencies in detecting changes in DoA or responses to noxious stimulation [3,44,72,73,157,173].

Because they depend on the frequency band of interest, SEF95 and SEF50 may demonstrate distinct sensitivity. For example, SEF95 outperformed SEF50 in predicting propofol-induced DoA in dogs [132] and showed greater sensitivity to noxious stimulation [94,95,140]. In contrast, SEF50 proved more effective in detecting nociceptive responses and their attenuation by analgesics in other studies [61,106,164,193].

Edge frequency interpretation becomes less reliable during periods of EEG suppression. Unpredictable high-frequency bursts and spikes can artificially elevate values, even as the anaesthetic depth increases [192,194]. To address this, correction formulas have been proposed to account for burst [194] and spike artefacts [195], and preliminary studies suggest improved performance when these are applied [116,117,118]. However, these correction methods remain simplistic and require further refinement.

Edge frequencies should not be viewed as linear indicators of anaesthetic depth but rather as context-dependent measures. Key questions for their interpretation include: Which edge frequency metric is used? What stage of DoA is being analysed? What EEG frequency transitions are likely occurring? Combining edge frequencies with complementary metrics—such as absolute band powers or power ratios—may enhance their utility in anaesthesia monitoring.

### 4.7. DoA Indices

The most frequently reported category of EEG-derived variables consisted of proprietary DoA indices, primarily developed for use in human patients. Among these, the BIS was the most prominent. These indices rely on combinations of EEG-derived variables—essentially, relative spectral powers and suppression ratio—processed through an algorithm to generate a scaled value, typically ranging from 0 (deep anaesthesia) to 100 (awake state). Although the algorithms behind commercial indices remain partially unpublished, their components and limitations are reported in the literature, including their application in veterinary anaesthesia [196,197].

Most studies demonstrated correlations between BIS values and anaesthetic dose, plasma concentration, clinical DoA assessments, or responses to noxious stimuli. However, notable interstudy variability in BIS measurements has been documented [105,110,111,151]. Moreover, BIS was often a poor predictor of movement responses to noxious stimulation [104,127]. At deeper levels of anaesthesia, BIS values become strongly correlated with SR, especially when SR exceeds 20–40%. This suggests that under such conditions, SR becomes the dominant contributing factor [34,198,199]. The proprietary nature of BIS algorithms poses a major limitation. The lack of transparency reduces interpretability and makes it difficult to identify potential biases in real-time monitoring. Other indices were not reported in enough studies to allow direct comparisons with BIS.

The patient state index was reported in pigs (*n* = 2) [3,170], horses (*n* = 2) [162,171], laboratory primates (*n* = 2) [153,165], and dogs (*n* = 1) [173]. The cerebral state index was recorded in six studies involving dogs [78,85,92,101,126,128]. The narcotrend index was used in sheep (*n* = 2) [125,147], cattle (*n* = 1) [123], horses (*n* = 1) [150], and cats (*n* = 1) [167]. Interestingly, one study assessing the patient state index across a wide DoA range (from awake to SR > 80%) reported a double-sigmoid pharmacodynamic relationship [3]. Similar patterns have been reported in humans for BIS and the narcotrend index [200,201,202,203]. This suggests that sensitivity to changes in the anaesthetic level varies across the DoA spectrum. These indices may exhibit a plateau phase, where intermediate changes in anaesthetic depth are no longer reflected in index values—particularly during surgical anaesthesia.

While these indices are among the most convenient variables for clinical use—being integrated into commercial EEG monitoring systems—they lack validation in animals. Their sensitivity across different DoA levels is limited, especially under surgical anaesthesia. Moreover, they fail to provide context-specific information to the clinician. The proprietary nature of their algorithms remains a barrier to advancing scientific understanding.

### 4.8. Entropy

Although entropy-based indices are seen as a promising alternative in human medicine, they have been reported in only a few animal studies. Response and state entropy indices offer great accessibility, as commercial devices (such as those from GE Healthcare) can easily measure them. In dogs, these entropy indices correlated with sevoflurane concentration and increased when a positive response was elicited by noxious stimulation at 1 MAC [143,160]. However, another study under similar conditions produced less conclusive results [115], highlighting variability in the reliability. In horses, entropy indices also correlated with the isoflurane concentration in a manner similar to BIS [166].

Several algorithms are available to calculate entropy measures from the EEG signal. While several methods are described in humans [204], veterinary studies have primarily utilised measures based on the Shannon entropy principle, which describes the irregularity, complexity, or unpredictability characteristics of the signal. Entropy can be applied to either the time domain EEG (temporal entropy) or the frequency domain spectrum (spectral entropy). State and response entropy are specific applications of the spectral entropy calculated over different frequency ranges, namely, 0.8 to 32 Hz and 0.8 to 47 Hz, respectively, with the latter including active EMG components [205]. In a study comparing several EEG-derived indices in dogs anaesthetised with propofol, temporal entropy demonstrated the best performance among the tested entropy and spectral measures [132]. Approximate entropy, a derivative of Shannon entropy [204], showed poor predictive performance for DoA in rabbits [117,118], and while it was applied in sheep, its effectiveness was not specifically investigated [84,89]. Permutation entropy has also been measured in dogs and rabbits; it quantifies EEG complexity by assessing the diversity of specific ordinal patterns in the EEG time series, with higher values reflecting more irregular, complex neural activity typical of lighter anaesthesia [206]. However, permutation entropy showed poor performance in evaluating DoA in dogs receiving propofol compared to temporal entropy [132].

In rabbits, permutation entropy showed good performance only at low propofol doses. Its predictive value improved markedly when corrections for burst suppression patterns were applied [117,118]. At DoA levels where bursts alternate with suppressed EEG epochs, entropy measurements become more difficult. In such cases, algorithmic adjustments are needed to preserve accuracy and reliability [204].

### 4.9. Graphical Display

In addition to specific metrics and indices, quantitative EEG-derived variables can be displayed graphically. Monitoring temporal changes in the spectrum across the full range of relevant frequencies requires analysing multiple relative power values and their ratios simultaneously. The compressed spectral array helps visualise these changes by displaying the power–frequency spectrum along the time axis [207]. This approach was used in two studies to compare EEG changes induced by different slaughtering methods in calves under light anaesthesia [95,97]. Although such visual analysis remains subjective, it allows for characterising the timing and duration of EEG pattern changes.

Because interpreting the three-dimensional graphic can be challenging, the density spectral array (DSA) improves readability by replacing the power axis with a colour scale. This method, known for several decades [208], has regained interest due to its ability to track real-time changes in the EEG frequency distribution with more precision and individualisation than commercial DoA indices [4]. Among the reviewed studies, eight used DSA. Six of these employed the Sedline^®^ commercial device to display the real-time DSA in experimental pigs receiving propofol [3,170], in chimpanzees given ketamine [165], and in canine [173] and equine patients [162,171] receiving multimodal anaesthesia. One earlier study in dogs presented post hoc DSA analysis using a custom-made routine coded in MATLAB (version R2022a/b) and raw EEG data from a diagnostic neurological device. It showed frequency-specific EEG changes in response to noxious stimulation during general anaesthesia [23]. Compared to recent developments in human medicine, the use and characterisation of DSA patterns and signatures in animals remain limited. Further investigation is needed across species, patient populations, and anaesthetic agents. Despite the current lack of detailed reference data, DSA offers a significant advantage over commercial indices by enabling more personalised, flexible, and comprehensive interpretation of EEG changes.

One study reported an alternative approach: instead of visualising absolute power, it applied the DSA to changes in power relative to a baseline epoch to better characterise variations induced by a noxious stimulation [172]. Although this is anecdotal, it illustrates how further exploration of DSA settings—such as referencing, filtering, and resolution—could enhance the monitoring of peri-anaesthetic EEG changes. While DSA is primarily a feature of commercial EEG-based DoA monitoring devices, these are constrained by proprietary sensors and software. However, an open-source, purpose-built MATLAB application has been used to generate real-time DSA in anaesthetised pigs, supporting the potential for future developments [209,210].

In conclusion, while displaying individual band power values and their ratios can improve sensitivity, DSA offers a powerful tool for clinicians. It allows simultaneous visualisation of relative changes in EEG frequency dominance, potentially enhancing real-time assessment of anaesthetic depth.

### 4.10. Other EEG Variables

Other EEG-derived variables were occasionally reported.

Amplitude-integrated EEG compresses the data into a trend display that shows peak-to-peak amplitude over extended periods. In newborn piglets, this method detected EEG depression induced by fentanyl [135] or Xenon [149], but it provided much less informative value than standard spectral metrics.

One study specifically reported power asymmetry, measuring the difference in EEG signals acquired simultaneously from left and right homologous electrode sites in dogs anaesthetised with isoflurane. However, no relevant results were found [10]. The asymmetry of the EEG signal between hemispheres has been poorly investigated during general anaesthesia.

The same study also assessed inter-hemispheric coherence, which measures the extent to which signals share common oscillatory activity and phase relationships at each frequency [10].

Another study plotted the magnitude of the bispectrum to investigate EEG changes associated with DoA and noxious stimulation in dogs [27]. Although bispectral analysis forms part of the widely used BIS index, it is rarely reported as a separate variable. Bispectral analysis is a higher-order frequency domain technique that quantifies both amplitude and phase relationships among different frequency components, allowing detection of nonlinear interactions and phase coupling that are not captured by conventional power spectral analysis [211]. Because bispectral analysis is a complex, three-dimensional process involving phase relationships between frequency bands, a more comprehensive bicoherence index is often calculated [212]. In dogs, the bicoherence index outperformed standard spectral variables for training a neural network to classify responders to noxious stimulation during halothane anaesthesia [27].

A similar approach was used with a neural network based on Lempel–Ziv complexity, which quantifies the degree of chaos in the EEG pattern. This method showed good predictive value in dogs under isoflurane anaesthesia [38]. Lempel–Ziv complexity is simple to compute and allows rapid real-time analysis. Although DoA assessment using this measure performed better than standard spectral metrics and has been occasionally reported in human studies, it was not found in other studies within this review.

In experimental primates, functional connectivity has been reported as a surrogate marker of awareness, distinguishing anaesthetised from awake status [161,213]. Functional connectivity measures the dynamic communication and synchronisation between different brain regions by assessing statistical relationships between their EEG signals. Anaesthesia typically reduces this connectivity, impairing large-scale information integration necessary for consciousness [214]. Although promising, functional connectivity analysis is complex and often requires advanced imaging techniques (e.g., functional magnetic resonance) or invasive acquisition methods, such as the high-definition cortical EEG.

Modulation of evoked EEG potentials by anaesthetic agents has also been used to characterise different anaesthetic states. Seven studies investigated the effect of DoA on the second differential of the middle latency of the auditory evoked potential (DD-MLAEP). This measure showed drug-specific correlations with concentrations of halothane, isoflurane, and methoxyflurane [30]. In horses under halothane anaesthesia, DD-MLAEP was reduced by ketamine infusion [35] and sarmazenil [55], but not by guaifenesin [40], midazolam [55], alfentanil [29], or thiopentone [41]. In young pigs, reversible CO2 anaesthesia significantly reduced the amplitude of the auditory evoked potential [51]. Somatosensory evoked potentials have also been evaluated to compare the effects of propofol and thiopentone [21], as well as Xenon [31], in cats. These methods require dedicated devices for data acquisition and analysis. They have not yet been sufficiently investigated for DoA assessment, but they may offer additional insights into the mechanisms and time course of anaesthesia-induced cerebral and nociceptive modulation.

## 5. Conclusions

In summary, a wide range of EEG-derived quantitative variables have been investigated for assessing the depth of anaesthesia in animals. The most frequently reported include spectral edge frequencies, spectral power metrics, suppression ratio, and proprietary indices such as BIS. While these measures provide valuable insights, their interpretation remains highly context-dependent. Even simple variables like suppression ratio, power ratios, or entropy require further research and refinement. Finally, although it is widely acknowledged that no single variable can reliably assess the depth of anaesthesia across the diversity of agents, doses, and species, little research has explored the benefits of combining multiple variables for improved accuracy and clinical usefulness.

## Figures and Tables

**Table 1:** Number of finally selected references according to descriptive subdivisions.

Divisions	Subdivisions	Number of References
Research topic “EEG used to evaluate levels of …”	Anaesthesia	129
	Sedation	5
	(Anti-)nociception	35
Animal species investigated	Canine	59
One paper reports the use	Equine	27
of two animal species (*)	Porcine *	24
	Rabbits	13
	Feline	10
	Ovine	10
	Avian	8
	Bovine	7
	Primate	6
	Caprine *	4
	Others (Dolphin, Deer)	2
EEG device	Laboratory device	66
Some papers report the use	Neurological diagnostic	18
of several devices	Aspect (BIS)	63
	Narcotrend	7
	Cerebral State Monitor	6
	Sedline	7
	IoC-View	3
	Datex entropy	4

**Table 2:** List of EEG-derived quantitative variables reported in the literature to evaluate depth of anaesthesia in animals and number of references (including the percentage over the total of *n* = 169).

Category	Number	Variables
Suppression ratio	44 (26%)	
Power	73 (43%)	Total power (*n* = 42) Absolute band power (*n* = 29) Relative band power (*n* = 16) Band power ratio (*n* = 10)
Edge frequency	74 (44%)	Edge 95% (*n* = 62) Edge 50% (Median, *n* = 60) Edge 90% (*n* = 6) Edge 80% (*n* = 5) Mean frequency (*n* = 2) Peak frequency (*n* = 2) Averaged instantaneous frequency (*n* = 1)
DoA index	76 (45%)	BIS index (*n* = 56) Patient State index (*n* = 7) Cerebral State Index (*n* = 6) Narcotrend index (*n* = 5) Index of Consciousness (*n* = 2)
Entropy	9 (5%)	Permutation entropy (*n* = 3) Approximate entropy (*n* = 4) Response entropy/State entropy (*n* = 4) Spectral entropy (*n* = 1) Temporal entropy (*n* = 1)
Visual graphy	10 (6%)	Compressed spectral array (*n* = 2) Density spectral array (*n* = 8)
Others	-	Auditory evoked potential (*n* = 7) Somatosensory evoked potential (*n* = 2) Amplitude integrated EEG (*n* = 2) Bicoherence index (*n* = 1) Bispectrum (*n* = 1) Functional connectivity per band (*n* = 1) Lempel–Ziv complexity (*n* = 1) Power asymmetry (*n* = 1) Spectral functional connectivity (*n* = 1) Waves per band (*n* = 1)

## Data Availability

The raw data supporting the conclusions of this article will be made available by the authors on request.

## Supplementary Materials

The following supporting information can be downloaded at: https://www.mdpi.com/article/10.3390/ani15152285/s1, Table S1: Keywords, MeSH terms, and search strings applied for the inclusion phase to extract from the PubMed database references on EEG-derived quantitative variables reported to evaluate depth of anaesthesia in animals (performed on 12 April 2023); Table S2: Keywords, MeSH terms, and search strings applied for the inclusion phase to extract from the Embase database references on EEG-derived quantitative variables reported to evaluate depth of anaesthesia in animals (performed on 12 April 2023); Table S3: Keywords, MeSH terms, and search strings applied for the inclusion phase to extract from the CAB Abstract database references on EEG-derived quantitative variables reported to evaluate depth of anaesthesia in animals (performed on 6 April 2023); Table S4: Final list of the selected references reporting EEG-derived quantitative variables to evaluate depth of anaesthesia in animals: Last name of the first author, Year of publication, Articles added during Stage 4 (Yes/No), Animal species investigated, EEG-derived variables reported, Level of evidence, Reference number in the main review article.

## Abbreviations

The following abbreviations are used in this manuscript:

BIS	Bispectral index
DD-MLAEP	Second derivative of the middle latency of the auditory evoked potential
DoA	Depth of anaesthesia
DSA	Density spectral array
EEG	Electroencephalography
MAC	Minimum alveolar concentration
PRISMA-ScR	Preferred Reporting Items for Systematic Reviews and Meta-Analyses extension for Scoping Reviews
PSI	Patient state index
SEF	Spectral edge frequency
SR	Suppression ratio

## References

[1] Moody O.A., Zhang E.R., Vincent K.F., Kato R., Melonakos E.D., Nehs C.J., Solt K. (2021). The neural circuits underlying general anesthesia and sleep. Anesth. Analg..

[2] Smajic J., Praso M., Hodzic M., Hodzic S., Srabovic-Okanovic A., Smajic N., Djonlagic Z. (2011). Assessment of depth of anesthesia: PRST score versus bispectral index. Med. Arh..

[3] Mirra A., Spadavecchia C., Levionnois O. (2022). Correlation of Sedline-generated variables and clinical signs with anaesthetic depth in experimental pigs receiving propofol. PLoS ONE.

[4] Purdon P.L., Sampson A., Pavone K.J., Brown E.N. (2015). Clinical electroencephalography for anesthesiologists: Part I: Background and basic signatures. Anesthesiology.

[5] Brown E.N., Purdon P.L., Akeju O., An J. (2018). Using EEG markers to make inferences about anaesthetic-induced altered states of arousal. Br. J. Anaesth..

[6] Campagnol D., Teixeira Neto F.J., Monteiro E.R., Beier S.L., Aguiar A.J. (2007). Use of bispectral index to monitor depth of anesthesia in isoflurane-anesthetized dogs. Am. J. Vet. Res..

[7] Lopes S., Rocha G., Guimaraes-Pereira L. (2024). Artificial intelligence and its clinical application in Anesthesiology: A systematic review. J. Clin. Monit. Comput..

[8] Murad M.H., Asi N., Alsawas M., Alahdab F. (2016). New evidence pyramid. Evid. Based Med..

[9] Greene S.A., Moore M.P., Keegan D., Gallagher L.V. (1991). Use of electroencephalographic monitoring for quantification of opioid or benzodiazepine antagonism in anaesthetized dogs. Proc. Assoc. Vet. Anaesth..

[10] Moore M.P., Greene S.A., Keegan R.D., Gallagher L., Gavin P.R., Kraft S.L., DeHaan C., Klappenbach K. (1991). Quantitative electroencephalography in dogs anesthetized with 2.0% end-tidal concentration of isoflurane anesthesia. Am. J. Vet. Res..

[11] Otto K.A., Short C.E. (1991). Cerebral responses in horses to halothane and isoflurane anaesthesia: EEG power spectrum analysis and differences in arteriovenous oxygen content. Proc. Assoc. Vet. Anaesth..

[12] Rampil I.J., Laster M., Dwyer R.C., Taheri S., Eger E.I. (1991). No EEG evidence of acute tolerance to desflurane in swine. Anesthesiology.

[13] Short C.E. (1991). Comparative cerebral responses to medetomidine at variable dosages with concurrent dobutamine or isoproterenol administration. Proc. Assoc. Vet. Anaesth..

[14] Artru A.A., Shapira Y., Bowdle T.A. (1992). Electroencephalogram, cerebral metabolic, and vascular responses to propofol anesthesia in dogs. J. Neurosurg. Anesthesiol..

[15] Greene S.A., Moore M.P., Keegan R.D., Gallagher L.V. (1992). Quantitative electroencephalography for measurement of central nervous system responses to diazepam and the benzodiazepine antagonist, flumazenil, in isoflurane-anaesthetized dogs. J. Vet. Pharmacol. Ther..

[16] Lanier W.L., Iaizzo P.A., Murray M.J. (1992). The effects of convective cooling and rewarming on systemic and central nervous system physiology in isoflurane-anesthetized dogs. Resuscitation.

[17] Ramani R., Todd M.M., Warner D.S. (1992). A dose-response study of the influence of propofol on cerebral blood flow, metabolism and the electroencephalogram in the rabbit. J. Neurosurg. Anesthesiol..

[18] Ekstrom P.M., Short C.E., Geimer T.R. (1993). Electroencephalography of detomidine-ketamine-halothane and detomidine-ketamine-isoflurane anesthetized horses during orthopedic surgery. A comparison. Vet. Surg..

[19] Keegan R.D., Greene S.A., Moore M.P., Gallagher L.V. (1993). Antagonism by flumazenil of midazolam-induced changes in quantitative electroencephalographic data from isoflurane-anesthetized dogs. Am. J. Vet. Res..

[20] Kochs E., Hoffman W.E., Werner C., Albrecht R.F., Schulte am Esch J. (1993). Cerebral blood flow velocity in relation to cerebral blood flow, cerebral metabolic rate for oxygen, and electroencephalogram analysis during isoflurane anesthesia in dogs. Anesth. Analg..

[21] Tomoda K., Shingu K., Osawa M., Murakawa M., Mori K. (1993). Comparison of CNS effects of propofol and thiopentone in cats. Br. J. Anaesth..

[22] Johnson C.B., Young S.S., Taylor P.M. (1994). Analysis of the frequency spectrum of the equine electroencephalogram during halothane anaesthesia. Res. Vet. Sci..

[23] Nayak A., Roy R.J., Sharma A. (1994). Time-frequency spectral representation of the EEG as an aid in the detection of depth of anesthesia. Ann. Biomed. Eng..

[24] Smith L.J., Greene S.A., Moore M.P., Keegan R.D. (1994). Effects of altered arterial carbon dioxide tension on quantitative electroencephalography in halothane-anesthetized dogs. Am. J. Vet. Res..

[25] Hartikainen K., Rorarius M., Makela K., Yli-Hankala A., Jantti V. (1995). Propofol and isoflurane induced EEG burst suppression patterns in rabbits. Acta Anaesthesiol. Scand..

[26] Miller S.M., Short C.E., Ekstrom P.M. (1995). Quantitative electroencephalographic evaluation to determine the quality of analgesia during anesthesia of horses for arthroscopic surgery. Am. J. Vet. Res..

[27] Muthuswamy J., Sharma A. (1996). A study of electroencephalographic descriptors and end-tidal concentration in estimating depth of anesthesia. J. Clin. Monit..

[28] Dougherty P.M., Li Y.J., Lenz F.A., Rowland L., Mittman S. (1997). Correlation of effects of general anesthetics on somatosensory neurons in the primate thalamus and cortical EEG power. J. Neurophysiol..

[29] Johnson C.B., Taylor P.M. (1997). Effects of alfentanil on the equine electroencephalogram during anaesthesia with halothane in oxygen. Res. Vet. Sci..

[30] Johnson C.B., Taylor P.M. (1998). Comparison of the effects of halothane, isoflurane and methoxyflurane on the electroencephalogram of the horse. Br. J. Anaesth..

[31] Utsumi J., Adachi T., Kurata J., Miyazaki Y., Shibata M., Murakawa M., Arai T., Mori K. (1998). Effect of xenon on central nervous system electrical activity during sevoflurane anaesthesia in cats: Comparison with nitrous oxide. Br. J. Anaesth..

[32] Antognini J.F., Carstens E. (1999). Isoflurane blunts electroencephalographic and thalamic-reticular formation responses to noxious stimulation in goats. Anesthesiology.

[33] Ghaly R.F., Lee J.J., Ham J.H., Stone J.L., George S., Raccforte P. (1999). Etomidate dose-response on somatosensory and transcranial magnetic induced spinal motor evoked potentials in primates. Neurol. Res..

[34] Haga H.A., Tevik A., Moerch H. (1999). Bispectral index as an indicator of anaesthetic depth during isoflurane anaesthesia in the pig. J. Vet. Anaesth..

[35] Johnson C.B., Bloomfield M., Taylor P.M. (1999). Effects of ketamine on the equine electroencephalogram during anesthesia with halothane in oxygen. Vet. Surg..

[36] Kral A., Tillein J., Hartmann R., Klinke R. (1999). Monitoring of anaesthesia in neurophysiological experiments. Neuroreport.

[37] Vachon P., Dupras J., Prout R., Blais D. (1999). EEG recordings in anesthetized rabbits: Comparison of ketamine-midazolam and telazol with or without xylazine. Contemp. Top. Lab. Anim. Sci..

[38] Zhang X.S., Roy R.J. (1999). Predicting movement during anaesthesia by complexity analysis of electroencephalograms. Med. Biol. Eng. Comput..

[39] Antognini J.F., Wang X.W., Carstens E. (2000). Isoflurane anaesthetic depth in goats monitored using the bispectral index of the electroencephalogram. Vet. Res. Commun..

[40] Johnson C.B., Bloomfield M., Taylor P.M. (2000). Effects of guaiphenesin on the equine electroencephalogram during anaesthesia with halothane in oxygen. Vet. Anaesth. Analg..

[41] Johnson C.B., Bloomfield M., Taylor P.M. (2000). Effects of thiopentone on the equine electroencephalogram during anaesthesia with halothane in oxygen. Vet. Anaesth. Analg..

[42] Schmidt M., Papp-Jambor C., Marx T., Schirmer U. (2000). Evaluation of bispectral index (BIS) for anaesthetic depth monitoring in pigs. Appl. Cardiopulm. Pathophysiol..

[43] Gavilanes A.W., Vles J.S., von Siebenthal K., Reulen J.P., Nieman F.H., van Sprundel R., Blanco C.E. (2001). Electrocortical brain activity, cerebral haemodynamics and oxygenation during progressive hypotension in newborn piglets. Clin. Neurophysiol..

[44] Haga H.A., Tevik A., Moerch H. (2001). Electroencephalographic and cardiovascular indicators of nociception during isoflurane anaesthesia in pigs. Vet. Anaesth. Analg..

[45] Itamoto K., Taura Y., Wada N., Taga A., Takuma T., Matsumura H., Miyara T. (2001). Effect of medetomidine on electroencephalography and use of a quantitative electroencephalograph for evaluating sedation levels in dogs. J. Vet. Med. A Physiol. Pathol. Clin. Med..

[46] Kruljc P. (2001). Beta, alpha, theta and delta brain waves and their proportions during ketamine- and halothane-induced anaesthesia in horses. Slov. Vet. Res..

[47] Otto K.A., Gerich T. (2001). Comparison of simultaneous changes in electroencephalographic and haemodynamic variables in sheep anaesthetised with halothane. Vet. Rec..

[48] Greene S.A., Benson G.J., Tranquilli W.J., Grimm K.A. (2002). Relationship of canine bispectral index to multiples of sevoflurane minimal alveolar concentration, using patch or subdermal electrodes. Comp. Med..

[49] Haga H.A., Dolvik N.I. (2002). Evaluation of the bispectral index as an indicator of degree of central nervous system depression in isoflurane-anesthetized horses. Am. J. Vet. Res..

[50] Itamoto K., Taura Y., Wada N., Takuma T., Une S., Nakaichi M., Hikasa Y. (2002). Quantitative electroencephalography of medetomidine, medetomidine-midazolam and medetomidine-midazolam-butorphanol in dogs. J. Vet. Med. A Physiol. Pathol. Clin. Med..

[51] Martoft L., Lomholt L., Kolthoff C., Rodriguez B.E., Jensen E.W., Jorgensen P.F., Pedersen H.D., Forslid A. (2002). Effects of CO2 anaesthesia on central nervous system activity in swine. Lab. Anim..

[52] Antognini J.F., Jinks S.L., Atherley R., Clayton C., Carstens E. (2003). Spinal anaesthesia indirectly depresses cortical activity associated with electrical stimulation of the reticular formation. Br. J. Anaesth..

[53] Bergamasco L., Accatino A., Priano L., Neiger-Aeschbacher G., Cizinauskas S., Jaggy A. (2003). Quantitative electroencephalographic findings in beagles anaesthetized with propofol. Vet. J..

[54] Greene S.A., Tranquilli W.J., Benson G.J., Grimm K.A. (2003). Effect of medetomidine administration on bispectral index measurements in dogs during anesthesia with isoflurane. Am. J. Vet. Res..

[55] Johnson C.B., Bloomfield M., Taylor P.M. (2003). Effects of midazolam and sarmazenil on the equine electroencephalogram during anaesthesia with halothane in oxygen. J. Vet. Pharmacol. Ther..

[56] Kruljc P., Cestnik V. (2003). Brain wave and electromyography responses to butorphanol during ketamine- and halothane-induced anaesthesia in horses. Acta Vet. Brno.

[57] March P.A., Muir W.W. (2003). Use of the bispectral index as a monitor of anesthetic depth in cats anesthetized with isoflurane. Am. J. Vet. Res..

[58] March P.A., Muir W.W. (2003). Minimum alveolar concentration measures of central nervous system activation in cats anesthetized with isoflurane. Am. J. Vet. Res..

[59] Martin-Cancho M.F., Lima J.R., Luis L., Crisostomo V., Ezquerra L.J., Carrasco M.S., Uson-Gargallo J. (2003). Bispectral index, spectral edge frequency 95%, and median frequency recorded for various concentrations of isoflurane and sevoflurane in pigs. Am. J. Vet. Res..

[60] Muir W.W., Wiese A.J., March P.A. (2003). Effects of morphine, lidocaine, ketamine, and morphine-lidocaine-ketamine drug combination on minimum alveolar concentration in dogs anesthetized with isoflurane. Am. J. Vet. Res..

[61] Murrell J.C., Johnson C.B., White K.L., Taylor P.M., Haberham Z.L., Waterman-Pearson A.E. (2003). Changes in the EEG during castration in horses and ponies anaesthetized with halothane. Vet. Anaesth. Analg..

[62] Otto K.A., Mally P. (2003). Noxious stimulation during orthopaedic surgery results in EEG ‘arousal’ or ‘paradoxical arousal’ reaction in isoflurane-anaesthetised sheep. Res. Vet. Sci..

[63] Trucchi G., Bergamasco L., Argento V. (2003). Intraoperative electroencephalographic monitoring: Quantitative analysis of bioelectrical data detected during surgical stimulation. Vet. Res. Commun..

[64] Carrasco-Jimenez M.S., Martin Cancho M.F., Lima J.R., Crisostomo V., Uson-Gargallo J., Ezquerra L.J. (2004). Relationships between a proprietary index, bispectral index, and hemodynamic variables as a means for evaluating depth of anesthesia in dogs anesthetized with sevoflurane. Am. J. Vet. Res..

[65] Greene S.A., Benson G.J., Tranquilli W.J., Grimm K.A. (2004). Effect of isoflurane, atracurium, fentanyl, and noxious stimulation on bispectral index in pigs. Comp. Med..

[66] Holmstrom A., Rosen I., Akeson J. (2004). Desflurane results in higher cerebral blood flow than sevoflurane or isoflurane at hypocapnia in pigs. Acta Anaesthesiol. Scand..

[67] Jang H.S., Kwon Y.S., Lee M.G., Jang K.H. (2004). The effect of tiletamine/zolazepam (Zoletile) combination with xylazine or medetomidine on electroencephalograms in dogs. J. Vet. Med. Sci..

[68] Lamont L.A., Greene S.A., Grimm K.A., Tranquilli W.J. (2004). Relationship of bispectral index to minimum alveolar concentration multiples of sevoflurane in cats. Am. J. Vet. Res..

[69] Luo A.L., Yi J., Guo X.Y., Ren H.Z., Huang Y.G., Ye T.H. (2004). Concentrations of propofol in cerebral spinal fluid: Target-controlled infusion. Chin. Med. J. (Engl.).

[70] Martin-Cancho M.F., Carrasco-Jimenez M.S., Lima J.R., Ezquerra L.J., Crisostomo V., Uson-Gargallo J. (2004). Assessment of the relationship of bispectral index values, hemodynamic changes, and recovery times associated with sevoflurane or propofol anesthesia in pigs. Am. J. Vet. Res..

[71] Culp L.B., Skarda R.T., Muir W.W. (2005). Comparisons of the effects of acupuncture, electroacupuncture, and transcutaneous cranial electrical stimulation on the minimum alveolar concentration of isoflurane in dogs. Am. J. Vet. Res..

[72] Haga H.A., Dolvik N.I. (2005). Electroencephalographic and cardiovascular variables as nociceptive indicators in isoflurane-anaesthetized horses. Vet. Anaesth. Analg..

[73] Haga H.A., Ranheim B. (2005). Castration of piglets: The analgesic effects of intratesticular and intrafunicular lidocaine injection. Vet. Anaesth. Analg..

[74] Johnson C.B., Stafford K.J., Sylvester S.P., Ward R.N., Mitchinson S., Mellor D.J. (2005). Effects of age on the electroencephalographic response to castration in lambs anaesthetised using halothane in oxygen. N. Z. Vet. J..

[75] Johnson C.B., Wilson P.R., Woodbury M.R., Caulkett N.A. (2005). Comparison of analgesic techniques for antler removal in halothane-anaesthetized red deer (*Cervus elaphus*): Electroencephalographic responses. Vet. Anaesth. Analg..

[76] Lamont L.A., Greene S.A., Grimm K.A., Tranquilli W.J. (2005). Relationship of feline bispectral index to multiples of isoflurane minimum alveolar concentration. Comp. Med..

[77] Murrell J.C., White K.L., Johnson C.B., Taylor P.M., Doherty T.J., Waterman-Pearson A.E. (2005). Investigation of the EEG effects of intravenous lidocaine during halothane anaesthesia in ponies. Vet. Anaesth. Analg..

[78] Bollen P.J., Saxtorph H. (2006). Cerebral state monitoring in beagle dogs sedated with medetomidine. Vet. Anaesth. Analg..

[79] Howard R.S., Finneran J.J., Ridgway S.H. (2006). Bispectral index monitoring of unihemispheric effects in dolphins. Anesth. Analg..

[80] Kruljc P., Nemec A. (2006). Electroencephalographic and electromyographic changes during the use of detomidine and detomidine-butorphanol combination in standing horses. Acta Vet. Hung..

[81] Kurita T., Morita K., Fukuda K., Takata K., Uraoka M., Sanjo Y., Sato S. (2006). Landiolol, an ultra-short-acting beta 1-adrenoceptor antagonist, does not alter the electroencephalographic effect of isoflurane in swine model. Br. J. Anaesth..

[82] Martin-Cancho M.F., Lima J.R., Luis L., Crisostomo V., Carrasco-Jimenez M.S., Uson-Gargallo J. (2006). Relationship of bispectral index values, haemodynamic changes and recovery times during sevoflurane or propofol anaesthesia in rabbits. Lab. Anim..

[83] Martin-Cancho M.F., Lima J.R., Luis L., Crisostomo V., Lopez M.A., Ezquerra L.J., Carrasco-Jimenez M.S., Uson-Gargallo J. (2006). Bispectral index, spectral edge frequency 95% and median frequency recorded at varying desflurane concentrations in pigs. Res. Vet. Sci..

[84] Voss L.J., Ludbrook G., Grant C., Sleigh J.W., Barnard J.P. (2006). Cerebral cortical effects of desflurane in sheep: Comparison with isoflurane, sevoflurane and enflurane. Acta Anaesthesiol. Scand..

[85] Bras S., Bressan N., Ribeiro L., Ferreira D.A., Antunes L., Nunes C.S. (2007). Nonlinear modeling of cerebral state index in dogs. Annu. Int. Conf. IEEE Eng. Med. Biol. Soc..

[86] Gibson T.J., Johnson C.B., Stafford K.J., Mitchinson S.L., Mellor D.J. (2007). Validation of the acute electroencephalographic responses of calves to noxious stimulus with scoop dehorning. N. Z. Vet. J..

[87] Kushiro T., Wiese A.J., Eppler M.C., Muir W.W. (2007). Effects of perzinfotel on the minimum alveolar concentration of isoflurane in dogs. Am. J. Vet. Res..

[88] Otto K.A. (2007). Effects of averaging data series on the electroencephalographic response to noxious visceral stimulation in isoflurane-anaesthetized dogs. Res. Vet. Sci..

[89] Voss L.J., Ludbrook G., Grant C., Upton R., Sleigh J.W. (2007). A comparison of pharmacokinetic/pharmacodynamic versus mass-balance measurement of brain concentrations of intravenous anesthetics in sheep. Anesth. Analg..

[90] Lopes P.C., Nunes N., Paula D.P., Nishimori C.T., Guerrero P.N., Conceicao E.D. (2008). Bispectral index in dogs at three intravenous infusion rates of propofol. Vet. Anaesth. Analg..

[91] Martin-Jurado O., Vogt R., Kutter A.P., Bettschart-Wolfensberger R., Hatt J.M. (2008). Effect of inhalation of isoflurane at end-tidal concentrations greater than, equal to, and less than the minimum anesthetic concentration on bispectral index in chickens. Am. J. Vet. Res..

[92] Ribeiro L.M., Ferreira D.A., Bressan N.M., Nunes C.S., Amorim P., Antunes L.M. (2008). Brain monitoring in dogs using the cerebral state index during the induction of anaesthesia via target-controlled infusion of propofol. Res. Vet. Sci..

[93] Tetrault S., Chever O., Sik A., Amzica F. (2008). Opening of the blood-brain barrier during isoflurane anaesthesia. Eur. J. Neurosci..

[94] Gibson T.J., Johnson C.B., Murrell J.C., Chambers J.P., Stafford K.J., Mellor D.J. (2009). Components of electroencephalographic responses to slaughter in halothane—Anaesthetised calves: Effects of cutting neck tissues compared with major blood vessels. N. Z. Vet. J..

[95] Gibson T.J., Johnson C.B., Murrell J.C., Hulls C.M., Mitchinson S.L., Stafford K.J., Johnstone A.C., Mellor D.J. (2009). Electroencephalographic responses of halothane—Anaesthetised calves to slaughter by ventral-neck incision without prior stunning. N. Z. Vet. J..

[96] Gibson T.J., Johnson C.B., Murrell J.C., Mitchinson S.L., Stafford K.J., Mellor D.J. (2009). Electroencephalographic responses to concussive non-penetrative captive-bolt stunning in halothane—Anaesthetised calves. N. Z. Vet. J..

[97] Gibson T.J., Johnson C.B., Murrell J.C., Mitchinson S.L., Stafford K.J., Mellor D.J. (2009). Amelioration of electroencephalographic responses to slaughter by non-penetrative captive-bolt stunning after ventral-neck incision in halothane—Anaesthetised calves. N. Z. Vet. J..

[98] Henao-Guerrero P.N., McMurphy R., Kukanich B., Hodgson D.S. (2009). Effect of morphine on the bispectral index during isoflurane anesthesia in dogs. Vet. Anaesth. Analg..

[99] Masamune T., Sato H., Okuyama K., Imai Y., Iwashita H., Ishiyama T., Oguchi T., Sessler D.I., Matsukawa T. (2009). The shivering threshold in rabbits with JM-1232(-), a new benzodiazepine receptor agonist. Anesth. Analg..

[100] Morgaz J., Granados M.M., Dominguez J.M., Navarrete R., Galan A., Fernandez J.A., Gomez-Villamandos R.J. (2009). Relationship of bispectral index to hemodynamic variables and alveolar concentration multiples of sevoflurane in puppies. Res. Vet. Sci..

[101] Ribeiro L.M., Ferreira D.A., Bras S., Castro A., Nunes C.A., Amorim P., Antunes L.M. (2009). Correlation between clinical signs of depth of anaesthesia and cerebral state index responses in dogs during induction of anaesthesia with propofol. Res. Vet. Sci..

[102] Ueyama Y., Lerche P., Eppler C.M., Muir W.W. (2009). Effects of intravenous administration of perzinfotel, fentanyl, and a combination of both drugs on the minimum alveolar concentration of isoflurane in dogs. Am. J. Vet. Res..

[103] Yamashita K., Akashi N., Katayama Y., Uchida Y., Umar M.A., Itami T., Inoue H., Sams R.A., Muir W.W. (2009). Evaluation of bispectral index (BIS) as an indicator of central nervous system depression in horses anesthetized with propofol. J. Vet. Med. Sci..

[104] Belda E., Blissitt K.J., Duncan J.C., Laredo F.G., Escobar Gil de Montes M., Clutton R.E. (2010). The bispectral index during recovery from halothane and sevoflurane anaesthesia in horses. Vet. Anaesth. Analg..

[105] Cavus E., Meybohm P., Doerges V., Hoecker J., Betz M., Hanss R., Steinfath M., Bein B. (2010). Effects of cerebral hypoperfusion on bispectral index: A randomised, controlled animal experiment during haemorrhagic shock. Resuscitation.

[106] Kongara K., Chambers J.P., Johnson C.B. (2010). Electroencephalographic responses of tramadol, parecoxib and morphine to acute noxious electrical stimulation in anaesthetised dogs. Res. Vet. Sci..

[107] Zwijnenberg R.J., del Rio C.L., Pollet R.A., Muir W.W. (2010). Effects of perzinfotel, butorphanol tartrate, and a butorphanol-perzinfotel combination on the minimum alveolar concentration of isoflurane in cats. Am. J. Vet. Res..

[108] Zwijnenberg R.J., del Rio C.L., Pollet R.A., Muir W.W. (2010). Effects of perzinfotel on the minimum alveolar concentration of isoflurane in dogs when administered as a preanesthetic via various routes or in combination with butorphanol. Am. J. Vet. Res..

[109] Ambrisko T.D., Johnson C.B., Chambers P. (2011). Effect of alfaxalone infusion on the electroencephalogram of dogs anaesthetized with halothane. Vet. Anaesth. Analg..

[110] Bleijenberg E.H., van Oostrom H., Akkerdaas L.C., Doornenbal A., Hellebrekers L.J. (2011). Bispectral index and the clinically evaluated anaesthetic depth in dogs. Vet. Anaesth. Analg..

[111] de Mattos-Junior E., Ito K.C., Conti-Patara A., de Carvalho Hda S., Reinoldes A., Caldeira Jde A., Cortopassi S.R. (2011). Bispectral monitoring in dogs subjected to ovariohysterectomy and anesthetized with halothane, isoflurane or sevoflurane. Vet. Anaesth. Analg..

[112] Haga H.A., Ranheim B., Spadavecchia C. (2011). Effects of isoflurane upon minimum alveolar concentration and cerebral cortex depression in pigs and goats: An interspecies comparison. Vet. J..

[113] Kazemi A., Harvey M., Cave G., Lahner D. (2011). The effect of lipid emulsion on depth of anaesthesia following thiopental administration to rabbits. Anaesthesia.

[114] Lopes P.C., Nunes N., Dias L.G.G.G., Pereira Neto G.B., Almeida R.M., Souza A.L.G., Belmonte E.A. (2011). Bispectral index in dogs with high intracranial pressure, anesthetized with propofol and submitted to two levels of FiO_2_. Arq. Bras. Med. Vet. E Zootec..

[115] Morgaz J., Granados Mdel M., Dominguez J.M., Navarrete R., Fernandez A., Galan A., Munoz P., Gomez-Villamandos R.J. (2011). Evaluation of spectral entropy to measure anaesthetic depth and antinociception in sevoflurane-anaesthetised beagle dogs. Vet. J..

[116] Otto K.A., Hoffler H.K., Cebotari S., Tudorache I. (2011). Relation between isoflurane concentration, body temperature and EEG variables during hypothermic cardiopulmonary bypass in juvenile sheep. Vet. J..

[117] Silva A., Campos S., Monteiro J., Venancio C., Costa B., Guedes de Pinho P., Antunes L. (2011). Performance of anesthetic depth indexes in rabbits under propofol anesthesia: Prediction probabilities and concentration-effect relations. Anesthesiology.

[118] Silva A., Ferreira D.A., Venancio C., Souza A.P., Antunes L.M. (2011). Performance of electroencephalogram-derived parameters in prediction of depth of anaesthesia in a rabbit model. Br. J. Anaesth..

[119] Belda E., Laredo F.G., Lucas X., Agut A., Escobar M., Soler M. (2012). The effects of atracurium on bispectral index (BIS) values in dogs anaesthetized with isoflurane. Vet. J..

[120] Benson E.R., Alphin R.L., Rankin M.K., Caputo M.P., Johnson A.L. (2012). Electroencephalogram-based methodology for determining unconsciousness during depopulation. Avian Dis..

[121] Benson E.R., Alphin R.L., Rankin M.K., Caputo M.P., Kinney C.A., Johnson A.L. (2012). Evaluation of EEG based determination of unconsciousness vs. loss of posture in broilers. Res. Vet. Sci..

[122] Kongara K., Chambers J.P., Johnson C.B. (2012). Effects of tramadol, morphine or their combination in dogs undergoing ovariohysterectomy on peri-operative electroencephalographic responses and post-operative pain. N. Z. Vet. J..

[123] Kulka A.M., Otto K.A., Bergfeld C., Beyerbach M., Kastner S.B. (2012). Effects of isoflurane anesthesia with and without dexmedetomidine or remifentanil on quantitative electroencephalographic variables before and after nociceptive stimulation in dogs. Am. J. Vet. Res..

[124] McIntosh M.P., Rajewski R.A. (2012). Comparative canine pharmacokinetics-pharmacodynamics of fospropofol disodium injection, propofol emulsion, and cyclodextrin-enabled propofol solution following bolus parenteral administration. J. Pharm. Sci..

[125] Otto K.A., Cebotari S., Hoffler H.K., Tudorache I. (2012). Electroencephalographic Narcotrend index, spectral edge frequency and median power frequency as guide to anaesthetic depth for cardiac surgery in laboratory sheep. Vet. J..

[126] Ribeiro L.M., Ferreira D.A., Bras S., Gonzalo-Orden J.M., Antunes L.M. (2012). Correlation between clinical signs of depth of anaesthesia and cerebral state index responses in dogs with different target-controlled infusions of propofol. Vet. Anaesth. Analg..

[127] Baars J.H., Rintisch U., Rehberg B., Lahrmann K.H., von Dincklage F. (2013). Prediction of motor responses to surgical stimuli during bilateral orchiectomy of pigs using nociceptive flexion reflexes and the bispectral index derived from the electroencephalogram. Vet. J..

[128] Bras S., Gouveia S., Ribeiro L., Ferreira D.A., Antunes L., Nunes C.S. (2013). Fuzzy logic model to describe anesthetic effect and muscular influence on EEG Cerebral State Index. Res. Vet. Sci..

[129] Ebner L.S., Lerche P., Bednarski R.M., Hubbell J.A. (2013). Effect of dexmedetomidine, morphine-lidocaine-ketamine, and dexmedetomidine-morphine-lidocaine-ketamine constant rate infusions on the minimum alveolar concentration of isoflurane and bispectral index in dogs. Am. J. Vet. Res..

[130] Kongara K., Chambers J.P., Johnson C.B., Dukkipati V.S. (2013). Effects of tramadol or morphine in dogs undergoing castration on intra-operative electroencephalogram responses and post-operative pain. N. Z. Vet. J..

[131] Saritas T.B., Saritas Z.K., Korkmaz M., Sivaci R.G. (2013). Comparison of bispectral index and vital parameters in rabbits receiving propofol or isoflurane anesthesia. Acta Sci. Vet..

[132] Bras S., Georgakis A., Ribeiro L., Ferreira D.A., Silva A., Antunes L., Nunes C.S. (2014). Electroencephalogram-based indices applied to dogs’ depth of anaesthesia monitoring. Res. Vet. Sci..

[133] Grint N.J., Johnson C.B., De Sa Lorena S., Luna S.P., Hussni C.A., Whay H.R., Murrell J.C. (2014). Electroencephalographic responses to a noxious surgical stimulus in mules, horses, and ponies. J. Equine Vet. Sci..

[134] McIlhone A.E., Beausoleil N.J., Johnson C.B., Mellor D.J. (2014). Effects of isoflurane, sevoflurane and methoxyflurane on the electroencephalogram of the chicken. Vet. Anaesth. Analg..

[135] Rey-Santano C., Mielgo V., Valls I.S.A., Encinas E., Lukas J.C., Vozmediano V., Suarez E. (2014). Evaluation of fentanyl disposition and effects in newborn piglets as an experimental model for human neonates. PLoS ONE.

[136] Romanov A., Moon R.S., Wang M., Joshi S. (2014). Paradoxical increase in the bispectral index during deep anesthesia in New Zealand white rabbits. J. Am. Assoc. Lab. Anim. Sci..

[137] Sandercock D.A., Auckburally A., Flaherty D., Sandilands V., McKeegan D.E. (2014). Avian reflex and electroencephalogram responses in different states of consciousness. Physiol. Behav..

[138] Saritas Z.K., Korkmaz M., Saritas T.B., Sivaci R.G. (2014). Comparison of the depth of anesthesia produced with dexmedetomidine-sevoflurane or medetomidine-sevoflurane by using bispectral index monitoring. Acta Sci. Vet..

[139] Terada Y., Ishiyama T., Asano N., Kotoda M., Ikemoto K., Shintani N., Sessler D.I., Matsukawa T. (2014). Optimal doses of sevoflurane and propofol in rabbits. BMC Res. Notes.

[140] Grint N.J., Johnson C.B., Clutton R.E., Whay H.R., Murrell J.C. (2015). Spontaneous electroencephalographic changes in a castration model as an indicator of nociception: A comparison between donkeys and ponies. Equine Vet. J..

[141] Jaber S.M., Sullivan S., Hankenson F.C., Kilbaugh T.J., Margulies S.S. (2015). Comparison of heart rate and blood pressure with toe pinch and bispectral index for monitoring the depth of anesthesia in piglets. J. Am. Assoc. Lab. Anim. Sci..

[142] Kaka U., Hui Cheng C., Meng G.Y., Fakurazi S., Kaka A., Behan A.A., Ebrahimi M. (2015). Electroencephalographic changes associated with antinociceptive actions of lidocaine, ketamine, meloxicam, and morphine administration in minimally anaesthetized dogs. Biomed. Res. Int..

[143] Mahidol C., Niyom S., Thitiyanaporn C., Suprasert A., Thengchaisri N. (2015). Effects of continuous intravenous infusion of morphine and morphine-tramadol on the minimum alveolar concentration of sevoflurane and electroencephalographic entropy indices in dogs. Vet. Anaesth. Analg..

[144] Verhoeven M.T., Gerritzen M.A., Kluivers-Poodt M., Hellebrekers L.J., Kemp B. (2015). Validation of behavioural indicators used to assess unconsciousness in sheep. Res. Vet. Sci..

[145] Kaka U., Goh Y.M., Chean L.W., Chen H.C. (2016). Electroencephalographic changes associated with non-invasive nociceptive stimulus in minimally anaesthetised dogs. Pol. J. Vet. Sci..

[146] Navarrete R., Quiros-Carmona S., Granados Mdel M., Gomez-Villamandos R.J., Dominguez J.M., Fernandez-Sarmiento J.A., Munoz-Rascon P., Funes F.J., Morgaz J. (2016). Effect of dexmedetomidine constant rate infusion on the bispectral index during alfaxalone anaesthesia in dogs. Vet. Anaesth. Analg..

[147] Otto K.A. (2016). Differential effects of propofol and isoflurane on the relationship between EEG Narcotrend index and clinical stages of anaesthetic depth in sheep undergoing experimental cardiac surgery. Vet. J..

[148] Qin B., Hu H., Cao B., Zhu Z. (2016). Effects of continuous infusion of etomidate at various dose rates on adrenal function in dogs. BMC Anesthesiol..

[149] Sabir H., Wood T., Gill H., Liu X., Dingley J., Thoresen M. (2016). Xenon depresses aEEG background voltage activity whilst maintaining cardiovascular stability in sedated healthy newborn pigs. J. Neurol. Sci..

[150] Tunsmeyer J., Hopster K., Kastner S.B. (2016). Clinical use of a multivariate electroencephalogram (Narcotrend) for assessment of anesthetic depth in horses during isoflurane-xylazine anesthesia. Front. Vet. Sci..

[151] Williams D.C., Aleman M.R., Brosnan R.J., Fletcher D.J., Holliday T.A., Tharp B., Kass P.H., Steffey E.P., LeCouteur R.A. (2016). Electroencephalogram of healthy horses during inhaled anesthesia. J. Vet. Intern. Med..

[152] Williams D.C., Brosnan R.J., Fletcher D.J., Aleman M., Holliday T.A., Tharp B., Kass P.H., LeCouteur R.A., Steffey E.P. (2016). Qualitative and quantitative characteristics of the electroencephalogram in normal horses during administration of inhaled anesthesia. J. Vet. Intern. Med..

[153] Zhang X., Liu S., Newport G.D., Paule M.G., Callicott R., Thompson J., Liu F., Patterson T.A., Berridge M.S., Apana S.M. (2016). In vivo monitoring of sevoflurane-induced adverse effects in neonatal nonhuman primates using small-animal positron emission tomography. Anesthesiology.

[154] Kells N.J., Beausoleil N.J., Chambers J.P., Sutherland M.A., Morrison R.S., Johnson C.B. (2017). Electroencephalographic responses of anaesthetized pigs (*Sus scrofa*) to tail docking using clippers or cautery iron performed at 2 or 20 days of age. Vet. Anaesth. Analg..

[155] Lehmann H.S., Musk G.C., Laurence M., Hyndman T.H., Tuke J., Collins T., Gleerup K.B., Johnson C.B. (2017). Mitigation of electroencephalographic and cardiovascular responses to castration in Bos indicus bulls following the administration of either lidocaine or meloxicam. Vet. Anaesth. Analg..

[156] McIlhone A.E., Beausoleil N.J., Kells N.J., Johnson C.B., Mellor D.J. (2018). Effects of halothane on the electroencephalogram of the chicken. Vet. Med. Sci..

[157] McIlhone A.E., Beausoleil N.J., Kells N.J., Mellor D.J., Johnson C.B. (2018). Effects of noxious stimuli on the electroencephalogram of anaesthetised chickens (*Gallus gallus domesticus*). PLoS ONE.

[158] Koyama C., Haruna T., Hagihira S., Yamashita K. (2019). New criteria of burst suppression on electroencephalogram in dogs anesthetized with sevoflurane. Res. Vet. Sci..

[159] Leitao C.J., Lima-Rodriguez J.R., Ferreira F., Avelino C., Sanchez-Margallo F.M., Antunes L. (2019). Parasympathetic tone activity evaluation to discriminate ketorolac and ketorolac/tramadol analgesia Level in swine. Anesth. Analg..

[160] Thengchaisri N., Mahidol C. (2019). Evaluating the effects of continuous intravenous infusions of tramadol and tramadol-lidocaine on sevoflurane minimum alveolar concentration (MAC) and entropy values in dogs. J. Vet. Med. Sci..

[161] Zhang Z., Cai D.C., Wang Z., Zeljic K., Wang Z., Wang Y. (2019). Isoflurane-induced burst suppression increases intrinsic functional connectivity of the monkey brain. Front. Neurosci..

[162] Drewnowska O., Turek B., Lisowska B., Short C.E. (2020). Preliminary study of the use of Root with Sedline® EEG monitoring for assessment of anesthesia depth in 6 Horses. Appl. Sci..

[163] Harris C., White P.J., Mohler V.L., Lomax S. (2020). Electroencephalography can distinguish between pain and anaesthetic intervention in conscious lambs undergoing castration. Animals.

[164] Karna S.R., Chambers P., Johnson C.B., Singh P., Stewart L.A., Lopez-Villalobos N., Kongara K. (2020). Effect of combinations of morphine, dexmedetomidine and maropitant on the electroencephalogram in response to acute electrical stimulation in anaesthetized dogs. J. Vet. Pharmacol. Ther..

[165] Mulreany L.M., Cushing A.C., Ashley A.L., Smith C.K. (2020). Potential for electroencephalographic monitoring of anesthetic depth in captive chimpanzees (*Pan troglodytes*) using a novel brain function monitor. J. Zoo Wildl. Med..

[166] Navarrete-Calvo R., Morgaz J., Gomez-Villamandos R.J., Quiros-Carmona S., Dominguez J.M., Ruiz-Lopez P., Granados M.M. (2020). Comparison of bispectral index and spectral entropy during isoflurane and medetomidine general anaesthesia in horses. Equine Vet. J..

[167] Raue J.F., Tunsmeyer J., Kastner S.B.R. (2020). Effects of isoflurane, remifentanil and dexmedetomidine on selected EEG parameters derived from a Narcotrend Monitor before and after nociceptive stimulation at different MAC multiples in cats. BMC Vet. Res..

[168] Velasco Gallego M.L., Martin Jurado O., Hatt J.M. (2021). Effects of isoflurane and sevoflurane alone and in combination with butorphanol or medetomidine on the bispectral index in chickens. BMC Vet. Res..

[169] Xie T., Chen K., Ma L., Ai Q., Liu Q., Hudson A.E. Brain connectivity analysis in anesthetized and awake states: An ECoG study in monkeys. Proceedings of the 2021 43rd Annual International Conference of the IEEE Engineering in Medicine & Biology Society (EMBC).

[170] Mirra A., Casoni D., Barge P., Hight D., Levionnois O., Spadavecchia C. (2022). Usability of the SedLine^®^ electroencephalographic monitor of depth of anaesthesia in pigs: A pilot study. J. Clin. Monit. Comput..

[171] Murillo C., Weng H.Y., Weil A.B., Kreuzer M., Ko J.C. (2022). Perioperative brain function monitoring with electroencephalography in horses anesthetized with multimodal balanced anesthetic protocol subjected to surgeries. Animals.

[172] Reiser J., Kreuzer M., Werner J., Saller A.M., Fischer J., Senf S., Deffner P., Abendschon N., Groll T., Grott A. (2022). Nociception-induced changes in electroencephalographic activity and FOS protein expression in piglets undergoing castration under isoflurane anaesthesia. Animals.

[173] Murillo C., Weil A.B., Moore G.E., Kreuzer M., Ko J.C. (2023). Electroencephalographic and cardiovascular changes associated with propofol constant rate of infusion anesthesia in young healthy dogs. Animals.

[174] Petrucci M., Spadavecchia C., Wanderer S., Boillat G., Marbacher S., Garcia Casalta L.G., Casoni D. (2023). Usefulness and reliability of the bispectral index during balanced anesthesia for neurovascular surgery in New Zealand White Rabbits. Brain Sci..

[175] Seddighi R., Geist A., Knych H., Sun X. (2023). The effect of remifentanil infusion on sevoflurane minimum alveolar concentration-no movement (MAC(NM)) and bispectral index in dogs. Vet. Anaesth. Analg..

[176] Zeng L., Brignardello-Petersen R., Guyatt G. (2021). When applying GRADE, how do we decide the target of certainty of evidence rating?. Evid. Based Ment. Health.

[177] Dixon-Woods M., Sutton A., Shaw R., Miller T., Smith J., Young B., Bonas S., Booth A., Jones D. (2007). Appraising qualitative research for inclusion in systematic reviews: A quantitative and qualitative comparison of three methods. J. Health Serv. Res. Policy.

[178] Hannes K., Lockwood C., Pearson A. (2010). A comparative analysis of three online appraisal instruments’ ability to assess validity in qualitative research. Qual. Health Res..

[179] Rampil I.J. (1998). A primer for EEG signal processing in anesthesia. Anesthesiology.

[180] Muhlhofer W.G., Zak R., Kamal T., Rizvi B., Sands L.P., Yuan M., Zhang X., Leung J.M. (2017). Burst-suppression ratio underestimates absolute duration of electroencephalogram suppression compared with visual analysis of intraoperative electroencephalogram. Br. J. Anaesth..

[181] Bruhn J., Bouillon T.W., Shafer S.L. (2001). Onset of propofol-induced burst suppression may be correctly detected as deepening of anaesthesia by approximate entropy but not by bispectral index. Br. J. Anaesth..

[182] Hirsch L.J., Fong M.W.K., Leitinger M., LaRoche S.M., Beniczky S., Abend N.S., Lee J.W., Wusthoff C.J., Hahn C.D., Westover M.B. (2021). American Clinical Neurophysiology Society’s Standardized Critical Care EEG Terminology: 2021 Version. J. Clin. Neurophysiol..

[183] Derbyshire A.J., Rempel B., Forbes A., Lambert E.F. (1936). The effects of anesthetics on action potentials in the cerebral cortex of the cat. Am. J. Physiol..

[184] Niedermeyer E. (2009). The burst-suppression electroencephalogram. Am. J. Electroneurodiagn. Technol..

[185] Park S.K., Han D.W., Chang C.H., Jung H., Kang H., Song Y. (2025). Association between intraoperative electroencephalogram burst suppression and postoperative delirium: A systematic review and meta-analysis. Anesthesiology.

[186] Hight D.F., Kaiser H.A., Sleigh J.W., Avidan M.S. (2020). Continuing professional development module: An updated introduction to electroencephalogram-based brain monitoring during intended general anesthesia. Can. J. Anaesth..

[187] Garcia P.S., Kreuzer M., Hight D., Sleigh J.W. (2021). Effects of noxious stimulation on the electroencephalogram during general anaesthesia: A narrative review and approach to analgesic titration. Br. J. Anaesth..

[188] Mirra A., Hight D., Spadavecchia C., Levionnois O.L. (2024). Spatio-temporal electroencephalographic power distribution in experimental pigs receiving propofol. PLoS ONE.

[189] Antognini J.F., Wang X.W., Carstens E. (1999). Quantitative and qualitative effects of isoflurane on movement occurring after noxious stimulation. Anesthesiology.

[190] Hight D., Ehrhardt A., Lersch F., Luedi M.M., Stuber F., Kaiser H.A. (2024). Lower alpha frequency of intraoperative frontal EEG is associated with postoperative delirium: A secondary propensity-matched analysis. J. Clin. Anesth..

[191] Caillet B., Maître G., Mirra A., Levionnois O.L., Simalatsar A. (2024). Measure of the prediction capability of EEG features for depth of anesthesia in pigs. Front. Med. Eng. Sec. Comput. Med..

[192] Tonner P.H., Bein B. (2006). Classic electroencephalographic parameters: Median frequency, spectral edge frequency etc. Best. Pract. Res. Clin. Anaesthesiol..

[193] Murrell J.C., Mitchinson S.L., Waters D., Johnson C.B. (2007). Comparative effect of thermal, mechanical, and electrical noxious stimuli on the electroencephalogram of the rat. Br. J. Anaesth..

[194] Rampil I.J., Lockhart S.H., Eger E.I., Yasuda N., Weiskopf R.B., Cahalan M.K. (1991). The electroencephalographic effects of desflurane in humans. Anesthesiology.

[195] Ihmsen H., Tzabazis A., Schywalsky M., Schwilden H. (2002). Propofol in rats: Testing for nonlinear pharmacokinetics and modelling acute tolerance to EEG effects. Eur. J. Anaesthesiol..

[196] March P.A., Muir W.W. (2005). Bispectral analysis of the electroencephalogram: A review of its development and use in anesthesia. Vet. Anaesth. Analg..

[197] Silva A., Antunes L. (2012). Electroencephalogram-based anaesthetic depth monitoring in laboratory animals. Lab. Anim..

[198] Bruhn J., Bouillon T.W., Shafer S.L. (2000). Bispectral index (BIS) and burst suppression: Revealing a part of the BIS algorithm. J. Clin. Monit. Comput..

[199] Morimoto Y., Hagihira S., Koizumi Y., Ishida K., Matsumoto M., Sakabe T. (2004). The relationship between bispectral index and electroencephalographic parameters during isoflurane anesthesia. Anesth. Analg..

[200] Kreuer S., Bruhn J., Larsen R., Grundmann U., Shafer S.L., Wilhelm W. (2004). Application of Bispectral Index and Narcotrend index to the measurement of the electroencephalographic effects of isoflurane with and without burst suppression. Anesthesiology.

[201] Kreuer S., Bruhn J., Ellerkmann R., Ziegeler S., Kubulus D., Wilhelm W. (2008). Failure of two commercial indexes and spectral parameters to reflect the pharmacodynamic effect of desflurane on EEG. J. Clin. Monit. Comput..

[202] Kreuer S., Bruhn J., Walter E., Larsen R., Apfel C.C., Grundmann U., Biedler A., Wilhelm W. (2008). Comparative pharmacodynamic modeling using bispectral and narcotrend-index with and without a pharmacodynamic plateau during sevoflurane anesthesia. Anesth. Analg..

[203] Han D.W., Linares-Perdomo O.J., Lee J.S., Kim J.H., Kern S.E. (2011). Comparison between cerebral state index and bispectral index as measures of electroencephalographic effects of sevoflurane using combined sigmoidal E(max) model. Acta Pharmacol. Sin..

[204] Schwilden H. (2006). Concepts of EEG processing: From power spectrum to bispectrum, fractals, entropies and all that. Best. Pract. Res. Clin. Anaesthesiol..

[205] Viertio-Oja H., Maja V., Sarkela M., Talja P., Tenkanen N., Tolvanen-Laakso H., Paloheimo M., Vakkuri A., Yli-Hankala A., Merilainen P. (2004). Description of the Entropy algorithm as applied in the Datex-Ohmeda S/5 Entropy Module. Acta Anaesthesiol. Scand..

[206] Olofsen E., Sleigh J.W., Dahan A. (2008). Permutation entropy of the electroencephalogram: A measure of anaesthetic drug effect. Br. J. Anaesth..

[207] Bickford R.G., Fleming N., Billinger T. (1971). Compression of EEG data. Trans. Am. Neurol. Assoc..

[208] Levy W.J., Shapiro H.M., Maruchak G., Meathe E. (1980). Automated EEG processing for intraoperative monitoring: A comparison of techniques. Anesthesiology.

[209] Caillet B., Devènes S., Maître G., Hight D., Mirra A., Levionnois O., Simalatsar A. (2023). General Anaesthesia Matlab-Based Graphical User Interface: A Tool for EEG Signal Acquisition, Processing and Visualisation Offline and in Real-Time. Technical Report. www.researchgate.net.

[210] Caillet B., Maître G., Devènes S., Hight D., Mirra A., Levionnois O.L., Simalatsar A. (2024). Long short-term-memory-based depth of anesthesia index computation for offline and real-time clinical application in pigs. Front. Med. Eng. Sec. Comput. Med..

[211] Sigl J.C., Chamoun N.G. (1994). An introduction to bispectral analysis for the electroencephalogram. J. Clin. Monit..

[212] Bouafif L. (2021). Monitoring of anesthesia by bispectral analysis of EEG signals. Comput. Math. Methods Med..

[213] Hudetz A.G. (2012). General anesthesia and human brain connectivity. Brain Connect..

[214] Chennu S., O’Connor S., Adapa R., Menon D.K., Bekinschtein T.A. (2016). Brain connectivity dissociates responsiveness from drug exposure during propofol-induced transitions of consciousness. PLoS Comput. Biol..

