# Supplementary material for: Quantitative Variables Derived from the Electroencephalographic Signal to Assess Depth of Anaesthesia in Animals: A Narrative Review
Source: Animals (Basel). 2025 Aug 5;15(15):2285. doi: 10.3390/ani15152285 (PMC12345460; doi:10.3390/ani15152285)
Supplement: Supplementary file 1 [file animals-15-02285-s001.zip › Table S4.pdf]

Table S4: Final list of the selected references reporting EEG-derived quantitative variables to evaluate depth of anaesthesia in animals: Last name of the first author, Year of publication, Articles added during Stage 4 (Yes/No), Animal species investigated, EEG-derived variables reported, Level of evidence, Reference number in the main review article.

ID	First author last name	Year	Stage 4	Species	Variables	Level	Ref.
1	Ambrisko	2011	Yes	Canine	P_freq, P_tot, Rel_P_band, SEF_50, SEF_95	M-3	[109]
2	Antognini	1999	Yes	Caprine	P_band, P_tot	M-3	[32]
3	Antognini	2000	No	Caprine	BIS, SEF_50, SEF_95	M-3	[39]
4	Antognini	2003	Yes	Caprine	BIS, SEF_95	M-3	[52]
5	Artru	1992	Yes	Canine	P_band, P_tot, SEF_95, Wave_band	M-3	[14]
6	Baars	2013	Yes	Porcine	BIS	M-2	[127]
7	Beldao	2010	Yes	Equine	BIS	M-2	[104]
8	Beldao	2012	Yes	Canine	BIS	M-2	[119]
9	Benson	2012	Yes	Avian	P_A/D	M-2	[120]
10	Benson	2012	Yes	Avian	P_A/D	M-2	[121]
11	Bergamasco	2003	Yes	Canine	P_A/D, P_B/D, P_band, P_T/D, Rel_P_band, SEF_50	M-2	[53]
12	Bleijenberg	2011	Yes	Canine	BIS	M-2	[110]
13	Bollen	2006	No	Canine	CSI, SR	M-3	[78]
14	Bras	2007	No	Canine	CSI	M-2	[85]
15	Bras	2013	Yes	Canine	CSI	M-2	[128]
16	Bras	2014	No	Canine	AIF, Perm_Ent, RPI, Spec_Ent, SEF_50, SEF_90, SEF_95, Temp_Ent	M-3	[132]
17	Campagnol	2007	No	Canine	BIS	M-2	[6]
18	Carrasco-Jimenez	2004	No	Canine	BIS	M-2	[64]
19	Cavus	2010	No	Porcine	BIS, SR	M-5	[105]
20	Culp	2005	No	Canine	BIS	S-2	[71]
21	de Mattos-Junior	2011	Yes	Canine	BIS	M-2	[111]
22	Dougherty	1997	No	Primate	P_band, P_tot, SEF_50, SEF_95	M-2	[28]
23	Drewnowska	2020	Yes	Equine	DSA, PSI	M-2	[162]
24	Ebner	2013	Yes	Canine	BIS	S-2	[129]
25	Ekstrom	1993	Yes	Equine	P_B/D, P_band, P_tot, SEF_95	M-2	[18]
26	Gavilanes	2001	No	Porcine	Rel_P_D	M-3	[43]
27	Ghaly	1999	No	Primate	SR	S-2	[33]
28	Gibson	2007	Yes	Cattle	P_tot, SEF_50, SEF_95	M-3	[86]
29	Gibson	2009	Yes	Cattle	CSA, P_tot, SEF_50, SEF_95	M-2	[94]
30	Gibson	2009	Yes	Cattle	CSA, P_tot, SEF_50, SEF_95	M-2	[95]

31	Gibson	2009	Yes	Cattle	CSA, P_tot, SR	M-2	[97]
32	Gibson	2009	Yes	Cattle	P_tot, SR	M-2	[96]
33	Greene	1991	No	Canine	P_band	M-2	[9]
34	Greene	1992	No	Canine	P_band	M-2	[15]
35	Greene	2002	No	Canine	BIS, SR	M-2	[48]
36	Greene	2003	No	Canine	BIS, SR	M-3	[54]
37	Greene	2004	No	Porcine	BIS, SR	M-3	[65]
38	Grint	2014	Yes	Equine	P_tot, SEF_50, SEF_95	M-2	[133]
39	Grint	2015	Yes	Equine	P_tot, SEF_50, SEF_95	M-2	[140]
40	Haga	1999	Yes	Porcine	BIS, SEF_95, SEF_50, SR	M-3	[34]
41	Haga	2001	No	Porcine	P_tot, P_A/D, P_B/D, P_T/D, P_tot, SEF_50, SEF_95, SR	M-3	[44]
42	Haga	2002	No	Equine	BIS	M-2	[49]
43	Haga	2005	No	Equine	P_A/D, P_B/D, P_T/D, P_tot, SEF_50, SEF_95, SR	M-2	[72]
44	Haga	2005	Yes	Porcine	P_A/D, P_B/D, P_band, P_tot, P_T/D, SEF_50, SEF_95	M-5	[73]
45	Haga	2011	Yes	Porcine<br>Caprine	SR	M-2	[112]
46	Harris	2020	No	Ovine	P_tot, SEF_50, SEF_95	M-3	[163]
47	Hartikainen	1995	Yes	Rabbit	SR	M-2	[25]
48	Henao-Guerrero	2009	Yes	Canine	BIS	M-2	[98]
49	Holmstrom	2004	No	Porcine	SR	S-2	[66]
50	Howard	2006	No	Dolphin	BIS	M-2	[79]
51	Itamoto	2001	Yes	Canine	P_band, SEF_50, SEF_90	M-3	[45]
52	Itamoto	2002	Yes	Canine	P_band, SEF_50, SEF_90	M-3	[50]
53	Jaber	2015	No	Porcine	BIS	M-3	[141]
54	Jang	2004	Yes	Canine	P_band	M-2	[67]
55	Johnson	1994	No	Equine	P_A/D, SEF_50, SEF_95	M-3	[22]
56	Johnson	1997	Yes	Equine	AEP, SEF_50, SEF_95	M-2	[29]
57	Johnson	1998	No	Equine	AEP, SEF_50, SEF_95	M-3	[30]
58	Johnson	1999	Yes	Equine	AEP, SEF_50, SEF_95	M-2	[35]
59	Johnson	2000	No	Equine	AEP, SEF_50, SEF_95	M-2	[41]
60	Johnson	2000	Yes	Equine	AEP, SEF_50, SEF_95	M-3	[40]
61	Johnson	2003	Yes	Equine	AEP, SEF_50, SEF_95	M-2	[55]
62	Johnson	2005	No	Ovine	P_tot, SEF_50, SEF_95	M-2	[74]
63	Johnson	2005	Yes	Deer	P_tot, SEF_50, SEF_95	M-2	[75]
64	Kaka	2015	Yes	Canine	P_tot, SEF_50	M-2	[142]
65	Kaka	2016	Yes	Canine	P_band, P_tot, SEF_50	M-2	[145]
66	Karna	2020	Yes	Canine	P_tot, SEF_50, SEF_95	M-3	[164]
67	Kazemi	2011	No	Rabbits	BIS	M-3	[113]

68	Keegan	1993	No	Canine	P_band	M-2	[19]
69	Kells	2017	Yes	Porcine	P_tot, SEF_50, SEF_95	M-2	[154]
70	Kochs	1993	Yes	Canine	P_band, P_tot, SEF_50, SR	S-2	[20]
71	Kongara	2010	Yes	Canine	P_tot, SEF_50, SEF_95	M-5	[106]
72	Kongara	2012	No	Canine	P_tot, SEF_50, SEF_95	M-3	[122]
73	Kongara	2013	No	Canine	P_tot, SEF_50, SEF_95	M-2	[130]
74	Koyama	2019	No	Canine	SR	M-3	[158]
75	Kral	1999	Yes	Feline	P_band	M-2	[36]
76	Kruljc	2001	No	Equine	Rel_P_Band	M-2	[46]
77	Kruljc	2003	No	Equine	Rel_P_Band, SEF_90	M-2	[56]
78	Kruljc	2006	No	Equine	Rel_P_Band, SEF_90	M-2	[80]
79	Kulka	2012	Yes	Cattle	Narcotrend_Index, P_band, Rel_P_band, SEF_50, SEF_95	M-3	[123]
80	Kurita	2006	No	Porcine	SEF_95	M-2	[81]
81	Kushiro	2007	No	Canine	BIS	M-2	[87]
82	Lamont	2004	Yes	Feline	BIS, SR	M-3	[68]
83	Lamont	2005	No	Feline	BIS, SR	M-3	[76]
84	Lanier	1992	No	Canine	P_band, SR	M-2	[16]
85	Lehmann	2017	Yes	Cattle	P_tot, SEF_50, SEF_95	M-3	[155]
86	Leitao	2019	No	Porcine	BIS	S-2	[159]
87	Lopes	2008	No	Canine	BIS	M-3	[90]
88	Lopes	2011	No	Canine	BIS, SR	M-2	[114]
89	Luo	2004	No	Canine	BIS	S-2	[69]
90	Mahidol	2015	Yes	Canine	Diff_Res_State_ent, Resp_ent, State_ent	S-2	[143]
91	March	2003	No	Feline	BIS	M-2	[58]
92	March	2003	No	Feline	BIS	M-3	[57]
93	Martin-Cancho	2003	Yes	Porcine	BIS	M-5	[59]
94	Martin-Cancho	2004	Yes	Porcine	BIS	M-2	[70]
95	Martin-Cancho	2006	Yes	Rabbit	BIS	M-2	[82]
96	Martin-Cancho	2006	Yes	Porcine	BIS, SEF_50, SEF_95	M-5	[83]
97	Martin-Jurado	2008	No	Avian	BIS, SR	M-3	[91]
98	Martoft	2002	No	Porcine	AEP, Rel_P_band, SEF_95, SR	M-2	[51]
99	Masamune	2009	Yes	Rabbit	BIS	S-2	[99]
100	McIlhone	2014	Yes	Avian	P_tot, Rel_P_band, SEF_50, SEF_95, SR	M-3	[134]
101	McIlhone	2018	Yes	Avian	P_tot, SEF_50, SEF_95, SR	M-3	[156]
102	McIlhone	2018	Yes	Avian	P_tot, SEF_50, SEF_95	M-3	[157]
103	McIntosh	2012	Yes	Canine	SEF_95	S-2	[124]
104	Miller	1995	No	Equine	P_B/D, P_tot, SEF_95	M-2	[26]
105	Mirra	2022	No	Porcine	DSA, P_band, PSI, SEF_95, SR	M-3	[170]
106	Mirra	2022	No	Porcine	DSA, P_band, PSI, SEF_95, SR	M-3	[3]

107	Moore	1991	Yes	Canine	Coherence, MF, P_assymetry, P_band, Peak_F, P_tot, SEF_50	M-2	[10]
108	Morgaz	2009	Yes	Canine	BIS	M-3	[100]
109	Morgaz	2011	Yes	Canine	Diff_Res_State_Entropy, Resp_Entropy, State_Entropy, SR	M-5	[115]
110	Muir	2003	No	Canine	BIS	M-2	[60]
111	Mulreany	2020	No	Primate	DSA, PSI, SR	M-2	[165]
112	Murillo	2022	No	Equine	DSA, PSI, SEF_95, Rel_P_band, SR	M-2	[171]
113	Murillo	2023	Yes	Canine	DSA, PSI, SEF_95, Rel_P_band, SR	M-3	[173]
114	Murrell	2003	No	Equine	P_tot, SEF_50, SEF_95	M-3	[61]
115	Murrell	2005	Yes	Equine	P_tot, SEF_50, SEF_95	M-3	[77]
116	Muthuswamy	1996	No	Canine	Bicoherence index, Bispectrum, SEF_50, SEF_90	M-3	[27]
117	Navarrete	2016	Yes	Canine	BIS, SR	M-2	[146]
118	Navarrete-Calvo	2020	No	Equine	BIS, Resp_Ent, State_Ent, SR	M-2	[166]
119	Nayak	1994	No	Canine	DSA	M-2	[23]
120	Otto	1991	Yes	Equine	P_A/D, P_B/D, P_T/D, Rel_P_band, SEF_80	M-2	[11]
121	Otto	2001	No	Ovine	P_A/D, P_B/D, P_T/D, Rel_P_band, SEF_50, SEF_80	M-3	[47]
122	Otto	2003	Yes	Ovine	P_A+T/D, Rel_P_band, SEF_50, SEF_80	M-2	[62]
123	Otto	2007	Yes	Canine	P_A/D, P_B/D, P_T/D, Rel_P_band, SEF_80, SEF_50	M-3	[88]
124	Otto	2011	Yes	Ovine	SEF_50, SEF_95, SR, SR_SEF_50, SR_SEF_95	M-2	[116]
125	Otto	2012	No	Ovine	Narcotrend_index, SEF_50, SEF_95	M-2	[125]
126	Otto	2016	No	Ovine	Narcotrend_index	M-2	[147]
127	Petrucci	2023	No	Rabbit	BIS, SR	M-2	[174]
128	Qin	2016	No	Canine	BIS	S-5	[148]
129	Ramani	1992	Yes	Rabbit	P_tot	M-2	[17]
130	Rampil	1991	No	Porcine	SR	M-3	[12]
131	Raue	2020	No	Feline	Narcotrend_index, SEF_50, SEF_95, SR	M-5	[167]
132	Reiser	2022	Yes	Porcine	DSA_change, P_tot	M-3	[172]
133	Rey-Santano	2014	No	Porcine	aEEG	S-2	[135]
134	Ribeiro	2008	No	Canine	CSI	M-3	[92]
135	Ribeiro	2009	No	Canine	CSI	M-3	[101]
136	Ribeiro	2012	Yes	Canine	CSI, SR	M-3	[126]
137	Romanov	2014	Yes	Rabbit	BIS, P_tot, SEF_95, SR	M-3	[136]
138	Sabir	2016	No	Porcine	aEEG	M-2	[149]

139	Sandercock	2014	Yes	Avian	P_tot, SEF_50, SEF_95	M-3	[137]
140	Saritas	2013	Yes	Rabbit	BIS	M-2	[131]
141	Saritas	2014	No	Rabbit	BIS	M-2	[138]
142	Schmidt	2000	No	Porcine	BIS	M-2	[42]
143	Seddighi	2023	No	Canine	BIS, SR	M-3	[175]
144	Short	1991	Yes	Canine	CSA, P_tot, Rel_P_band	M-2	[13]
145	Silva	2011	No	Rabbit	Approx_ent, CMSPE, IoC, Perm_ent, SEF_50, SEF_95, SR, SR_Perm_Ent, SR_SEF_50, SR_SEF_95	M-5	[117]
146	Silva	2011	No	Rabbit	Approx_ent, CMSPE, IoC, Perm_ent, SEF_50, SEF_95, SR, SR_Perm_Ent, SR_SEF_50, SR_SEF_95	M-2	[118]
147	Smith	1994	No	Canine	P_band	M-2	[24]
148	Terada	2014	Yes	Rabbit	BIS	M-2	[139]
149	Tetrault	2008	No	Feline	P_band	M-2	[93]
150	Thengchaisri	2019	Yes	Canine	Diff_Res_State_ent, Resp_ent, State_ent	M-3	[160]
151	Tomoda	1993	No	Feline	SEP	M-3	[21]
152	Trucchi	2003	No	Canine	MF, P_band, Peak_F, SEF_50	M-2	[63]
153	Tunsmeyer	2016	No	Equine	Narcotrend_Index	M-5	[150]
154	Ueyama	2009	No	Canine	BIS	M-2	[102]
155	Utsumi	1998	No	Feline	SEP	M-2	[31]
156	Vachon	1999	No	Rabbit	P_B/D, P_band, SEF_80, SEF_95	M-3	[37]
157	Velasco Gallego	2021	No	Avian	BIS, SR	M-3	[168]
158	Verhoeven	2015	Yes	Ovine	P_band, P_tot, SEF_95	M-2	[144]
159	Voss	2006	No	Ovine	Approx_ent	M-2	[84]
160	Voss	2007	Yes	Ovine	Approx_ent	S-2	[89]
161	Williams	2016	No	Equine	BIS, P_band, P_tot, SEF_50, SEF_95, SR	M-3	[151]
162	Williams	2016	Yes	Equine	BIS, P_band, P_tot, SEF_50, SEF_95, SR	M-2	[152]
163	Xie	2021	No	Primate	Functional_connectivity_band	M-3	[169]
164	Yamashita	2009	Yes	Equine	BIS	M-3	[103]
165	Zhang	1999	Yes	Canine	Lempel-Ziv complexity, SEF_50, SEF_95	M-2	[38]
166	Zhang	2016	No	Primate	PSI	S-2	[153]
167	Zhang	2019	No	Primate	Functional_connectivity_tot	M-2	[161]
168	Zwijnenberg	2010	No	Feline	BIS	S-2	[107]
169	Zwijnenberg	2010	No	Canine	BIS	S-2	[108]

Level of Evidence: M: Main/Primary objective, S: Secondary objective, 1: Case series/Case report, 2: Observational study, 3: Case control study, 4: Cohort study, 5: Randomised controlled trial, 6: Systematic review/Meta-analysis; AEP: Auditory evoked potential; AIF: Averaged instantaneous frequency; aEEG: Amplitude-integrated EEG; Approx_Entropy: Approximate entropy; BIS: Bispectral index; CMSPE: Composite multiscale permutation entropy; CSI: Cerebral state index; CSA: Condensed spectral array; Diff_Res_State_Entropy: Difference between response and state entropy; DSA: Density spectral array; Functional_connectivity_tot: Functional connectivity of the total power; IoC: Index of Consciousness; MF: Mean frequency; P_A/D: Ratio of the alpha over delta power; P_A+T/D: ratio of the alpha + theta over delta power; P_B/D: Ratio of the beta over deta power; P_band: Power per bandwidth; P_assymetry: Power assymetry; P_freq: Power per frequency integer; P_T/D: Ratio of the theta over delta power; P_tot: Total power; Peak_F: Peak frequency; Perm_Entropy: Permutation entropy; PSI: Patient state index; Rel_P_band: Relative power per bandwidth; Rel_P_D: relative delta power; Resp_Entropy: response entropy; RPI: Relative power index; SEF_50: Spectral edge frequency 50% (Median frequency); SEF_80: Spectral edge frequency 80%; SEF_90: Spectral edge frequency 90%; SEF_95: Spectral edge frequency 95%; SEP: Somatosensory evoked potential; Spec_Entropy: Spectral entropy; SR: Suppression ratio; SR_*: SR-compensated; State_Entropy: State entropy; Temp_Entropy: Temporal entropy; Wave_band: number of waves per bandwidth.
